# Modelling Circulating Tumour Cells for Personalised Survival Prediction in Metastatic Breast Cancer

**DOI:** 10.1371/journal.pcbi.1004199

**Published:** 2015-05-15

**Authors:** Gianluca Ascolani, Annalisa Occhipinti, Pietro Liò

**Affiliations:** University of Cambridge, Computer Laboratory, Cambridge, United Kingdom; University of California-Irvine, UNITED STATES

## Abstract

Ductal carcinoma is one of the most common cancers among women, and the main cause of death is the formation of metastases. The development of metastases is caused by cancer cells that migrate from the primary tumour site (the mammary duct) through the blood vessels and extravasating they initiate metastasis. Here, we propose a multi-compartment model which mimics the dynamics of tumoural cells in the mammary duct, in the circulatory system and in the bone. Through a branching process model, we describe the relation between the survival times and the four markers mainly involved in metastatic breast cancer (EPCAM, CD47, CD44 and MET). In particular, the model takes into account the gene expression profile of circulating tumour cells to predict personalised survival probability. We also include the administration of drugs as bisphosphonates, which reduce the formation of circulating tumour cells and their survival in the blood vessels, in order to analyse the dynamic changes induced by the therapy.

We analyse the effects of circulating tumour cells on the progression of the disease providing a quantitative measure of the cell driver mutations needed for invading the bone tissue. Our model allows to design intervention scenarios that alter the patient-specific survival probability by modifying the populations of circulating tumour cells and it could be extended to other cancer metastasis dynamics.

## Author Summary

Breast cancer is caused by genetic mutations leading to uncontrollable cell reproduction. During successive proliferations, the progenies of tumour cells acquire further mutations increasing their heterogeneity. Among the tumoural mutated cells, there are some which present specific markers of increased aggressiveness and resistance. Sufficiently skilled cancer cells detach from the mammary epithelial cells, enter the blood vessels becoming circulating tumour cells, and reach the bone tissue where they seed. Breast cancer survival probability is the statistical representation of clinical data describing the times patients will survive after the diagnosis of the disease. Breast cancer survival is strongly correlated to genetic markers which increase the resistance and the invading skills of cancer cells but, it is poorly correlated to the amount of circulating tumour cells. To improve the understanding of the dynamic progression of the disease and assisting biologists in the interpretation of results and in experimental design, we developed a mathematical model encompassing the evolution of cancer cells originated in the breast, passing through the circulatory system, and invading the bone tissue based on survival probabilities of patients with different genetic expressions. The model allows us to strongly correlate the gene expression data of cancer cells with the survival probability by identifying the circulating tumour cells responsible for the formation of metastasis. Survival probabilities generated with the model are a useful tool to identify the presence of hidden markers not yet taken into consideration and study the effects of drugs’ administration.

## Introduction

Breast cancer is characterised by multi-year survival from the first diagnosis of bone metastases. It is a leading cause of cancer death among women, and if detected at an early stage, its prognosis is favourable, with 5-year survival—for death from the cancer—in more than 90% of the patients. However, when initial diagnosis is of advanced metastatic disease, the 5-year survivals decrease to around 30%. The survival and prognosis of cancer patients with metastatic skeletal disease vary widely and depend on many factors including features of the primary tumour (histological type and grade), presence of extraosseus metastatic disease, patient’s characteristic (performance status and age), level of tumour markers and extension of skeletal disease. In fact, every cancer is different; as cancer grows, a mixture of cells builds up over time and becomes more and more complex. Cancer cells often detach from the primary tumour, become circulating tumour cells (CTC) and invade blood vessels. Once in the bloodstream, they reach the skeleton and adhering to the endosteal surface, they colonize the bone, subverting the cellular processes of normal remodelling and causing bone pathology [1]. Cancer phenotypic heterogeneity may be due to progressive, but asynchronous changes in tumour–bone interactions (i.e. progressive accumulation of driver and non driver mutations). In particular, the Transforming Growth Factor-*β* (TGF-*β*) pathway mutations are determinant in generating cancer heterogeneity and in the formation of CTCs causing bone metastasis. TGF-*β* is among the most abundant growth factors in bone, and its role in skeletal metastases is established. It is deposited in the bone matrix by osteoblasts, released and activated during osteoclastic resorption, and it regulates bone development and remodelling [2]. Advanced cancers frequently escape growth inhibition by TGF-*β*, which also activates epithelial-mesenchymal transition (EMT) and invasion, promoting metastases. TGF-*β* also increases angiogenesis and suppresses immune surveillance. It specifically stimulates bone metastases by inducing pro-osteolytic gene expression in cancer cells, such as parathyroid hormone related protein (PTHrP) [3]. Moreover, therapies acting on the TGF-*β* pathway seem effective at all levels and compartments where TGF-*β* is involved, generating a retroaction effects on the primary tumour, the circulatory system and the bone [4].

Recently, Baccelli et al. [5] have identified a set of genetic markers in CTCs which are key players in establishing bone metastasis (metastasis-initiating cells) and largely influencing outcomes and patient’s survival. The overexpression of EPCAM, CD44, CD47 and MET cell proteins in a subset of CTCs correlates with lower overall survival. These four markers are known to be involved in tumourigenesis [6–8] and are co-regulated with the TGF-*β* signalling pathway [9].

Because of the complexly structured and heterogeneous process as well as the paucity of experimental data, it is important to model how the dynamics of TGF-*β*-driven CTCs couples with the primary (mammary duct) and secondary (bone niche) cancers. Indeed, cancer mathematical models play an important role in assisting biologists in the interpretation of results and in experimental design (see Maini [10], Bellomo [11] and Chaplain [12] for breast cancer and bone cancer modelling, among others) with a growing interest in combining epidemiological (e.g. survival information), clinical and molecular data.

**Fig 1:**
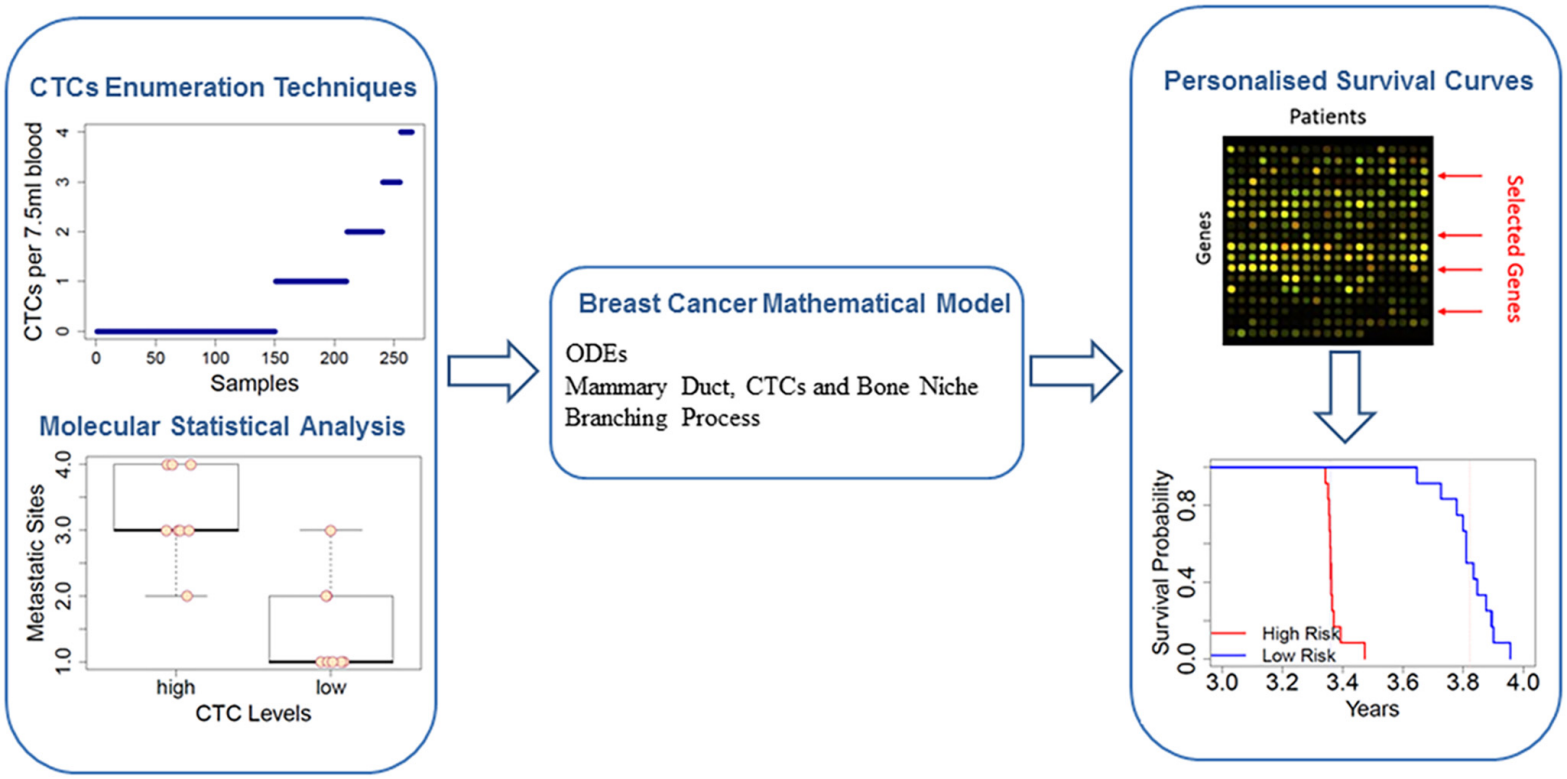
Overview of the Breast Cancer Model. Starting from molecular and clinical data on CTCs levels, we model the evolution of cancer cells from the primary tumour (mammary duct) to the formation of metastasis (in the bone niche). By using the model, we predict the survival probability in metastatic breast cancer patients under different conditions (particular gene expression profiles or different response of the immune system).

In a recent work [2], we have modeled how TGF-*β* drives the formation of early neoplastic signature in breast and perturbs the bone remodelling process. Here, we present a multi-compartment mathematical model that aims at elucidating the effects of the TGF-*β* and the concomitant therapies in the three microenvironments (mammary duct, circulatory system and bone niche). Fig 1 summarises the structure of the model. Starting from statistical data (including molecular and clinical data), we develop a model able to predict the survival probability by using the gene expression profile of CTCs. We aim at a quantitative understanding of the relationship between gene expression levels in breast cancer and formation of bone metastasis with respect to the survival statistics. Indeed, we propose a mathematical model linking the amount of CTCs to the survival times, in order to predict the patient-specific survival. By using a branching process technique [13], we compute the probability of developing *EPCAM* ^+^ *CD*44^+^ *MET* ^+^ *CD*47^+^ CTCs. Through the model it is also possible to predict bisphosphonates-therapy outcomes based on the patient’s specific markers. Bisphosphonates are drugs commonly used as treatment for several bone diseases in order to reduce osteoporosis and recent works have shown the anti-tumour effectiveness of bisphosphonates administered in a biological window therapy in naive bone-only metastatic and locally advanced breast cancer [14] (see also [15]).

This work is organised in the following way: in the first two subsections of the Results section, we discuss the roles of TGF-*β* and CTCs in metastatic breast cancer. In the third subsection, we present the system of ODEs for each compartment (mammary duct and circulatory system). The equations including the treatment are reported in forth subsection. The fifth subsection shows how we used the model to simulate the disease evolution so to produce survival curves. In the sixth subsection, we present the results obtained by numerical simulations and we discuss the cases of higher number of driver mutations and the case of immune response delay. Information relative to the analysis of gene expression data is given in Methods section. Finally, the conclusions give a brief summary and critique of the findings.

## Results

### Breast cancer: the relationship between TGF-*β* and circulating tumour cells

The earliest stage of breast cancer is revealed by abnormal cells inside a ductal lobular unit in the breast. In these cells, the TGF-*β* is highly expressed and induces cells to undergo apoptosis. Recent studies about TGF-*β* activation have highlighted the important role of integrins, an adhesion molecule that mediates the attachment between a cell and its surroundings [16]. In particular, the binding of integrins to the latent TGF-*β* promotes the production of active TGF-*β*. The invasive ductal carcinoma is characterised by the loss of epithelial cadherin (E-cad) function via epigenetic silencing, or via genetic inactivation by TGF-*β* [17]. E-cad is a hallmark of well differentiated epithelium, and it maintains the junction between cells preventing the cancer cell proliferation and migration. Indeed, the E-cad downregulation by TGF-*β* is proved to prevent mammary cell differentiation and produces more spherical cells which promote metastatic growth [18].

### Circulating tumour cells: their source and their survival

Cells with driver mutations causing TGF-*β* resistance, when located close to sites of elevated activation of the transforming growth factor, diminish their E-cad induced adhesion and reduce the probability of incurring death [18]. Amongst all the mutated cells, only those freed from the cell-to-cell junctions have a higher possibility of migrating through the TGF-*β* altered tissue and reaching the near blood capillaries. Therefore, CTCs are cells originated from the primary tumour site (and/or secondary metastatic sites) and sharing the same characteristics and the same phenotype heterogeneity of the primary tumour cells. In breast cancer, the epithelial cells in the mammary ducts affected by the tumour represent the main sources of CTCs [19]. Moreover, the size of the tumour is a sort of power source factor that contributes to the quantity of CTCs in the blood stream, so that bigger tumour sizes correspond to higher numbers of CTCs. Different types of breast cancer affect the power source as a result of their different velocity in evolving and growing throughout the tissue. On the other hand, we assume that in tumours of the same size and with the same rate of E-cad unbinding, CTC sources behave equally (i.e. they release the same amount of CTCs per unit of time). The explanation is given by the fact that only when a cell is completely separated from the surrounding neighbour cells and the extracellular matrix, it can be part of the amount of CTCs.

CTCs is associated with large quantities of TGF-*β* as well as their progenitors [20]. The synthesised TGF-*β*, which depends on the cell phenotypes, might not suffice the cancer cells’ need because of the more dispersive space and less cramped geometry which facilitate the dispersion of TGF-*β*. Nevertheless, TGF-*β* production serves also as an alerting inflammatory signal helping the immune system to detect and attack the CTCs. In part for their instabilities caused by mutations and in part for the immune system response, generally CTCs do not survive long in the blood stream. This is true especially when the concentration of CTCs is low while, at higher concentration, CTCs cluttering and overwhelming of the immune system might extend the life of the same cancer cells [21]. Furthermore, CTCs seem to have very low proliferation rate when flowing in the blood stream.

Using different technological platforms, clusters of CTCs has been detected within the circulating system of patients with cancers of different origin [21]. While most clusters are relatively small, ranging from 2 to 50 cancer cells, they have from 23- to 50-fold increased metastatic potential. This property of CTC clusters, together with the adverse prognosis of breast cancer patients with abundant CTCs clusters, support an important role for these cells in the blood-bone spread of cancer.

It has been experimentally shown that CTCs overexpressing EPCAM, CD47, CD44 and MET have a high probability of succeeding in generating bone metastasis [5]. Overexpression of EPCAM is a phenotypical characteristic inherited since the tumour cell was in the lobular duct and remained present during the epithelial-mesenchymal transition (EMT) process. In the extravasation process, EPCAM helps cancer cells in exiting the circulatory system, by inducing the anchorage between CTCs and the vascular endothelium [7, 8, 22].

CD47 is a protein expressed on all the cell membranes, and it interacts with integrins and immunogenic complexes on the cells. It is involved in several processes, including the spreading and aggregation of platelets [23], and modulation of T-cell activation [24, 25]. CD47 operates as a “self” marker on red blood cells in order to prevent their clearance by macrophages [26]. Elevated expression of CD47 helps CTCs to evade the immune system.

CD44 is a receptor principally present on lymphocytes. This protein is implicated in a variety of immunological functions, such as vascular extravasation and T-cell co-stimulation [27]. CD44 is prevalently upregulated at various stages of the cancer evolution, and the protein also mediates adhesion between stroma cells and bone marrow progenitor cells. Promotion of CTCs extravasation across endothelial vessels and homing into peripheral organs makes CD44 responsible for metastasis formation in the bone tissue [28–31].

MET is a receptor involved in embryonic development and organ regeneration. It contributes to establish the normal tissue patterning by orchestrating cell proliferation, disrupting the cell-to-cell junctions, facilitating the migration through the extracellular matrix and inhibiting apoptosis [6]. MET deregulation induces cancer cells to leave the primary tumour, move towards different organs and give rise to metastasis [32]. In the long run, the heterogeneity of CTCs will increase reflecting the phenotypic cellular diversity in the primary tumour source. At the same time, a small component of the whole CTC population capable of evading the immune system (*EPCAM* ^+^ *CD*47^+^ *CD*44^+^ *MET* ^+^ CTCs), extravasating and seeding in the bone will branch from the rest of the CTCs and initiate the process of development of metastasis.

**CTCs and bone metastasis formation.** The CTC populations have different phenotypes reflecting the heterogeneity of the primary tumour source. Among different cancer cells, those with a phenotype more sensitive to the TGF-*β* chemoattraction reach the bone niche. Furthermore, during the bone remodelling process, TGF-*β* and cytokines attract the near blood vessels toward the portion of lesioned bone matrix. The reduced distance between the peripheral blood stream and the source of TGF-*β* increases the probability of few CTCs exiting the capillary and entering the bone tissue. CTCs in the fractured bone rarely begin a fast invasion of the tissue, on the other hand, they change the remodelling process of the bone by strongly interfering with the quantity of TGF-*β* involved in the differentiation and maturation of the osteoblasts and cause a prolonged osteolytic activity. Cancer cells provoke a reduction of bone re-mineralization which results in a weaker bone, hence higher probability of re-occurrence of new fracture-remodelling cycles. Meanwhile, the number of CTCs slowly increases taking advantage of the extra TGF-*β* released and extra space left in the bone multicellular unit (BMU) at each cycle.

### Multi-compartment mathematical model

In order to describe the early stages of breast cancer and the formation of metastasis, we develop a model that includes three compartments representing three distinct body systems and involves different regions of the body: 1) the epithelial tissue in the mammary duct, 2) the circulatory system and 3) the bone. Our approach, even though it encompases extense and distinct body parts, allows us to semiquantitatively reproduce the progression of the disease.

The first and the third compartments are geometrically connected through the circulatory system which plays a fundamental role in the migration of cancer cells from the primary tumour site in the mammary duct to the secondary sites in the bone tissue. Hence, the three compartments are kinematically and dynamically interconnected. The model shows the evolution of the tumour in terms of invasion of the three compartments by cancer cells. The different fitness landscapes of cancer cells surviving in each compartment (cancer cells intravasation, extravasation and metastasis formation) represent the interactions between the cancer cells and the environment.

We constrained ourselves to the early stages of breast cancer for the sake of simplicity. During the early stages, the concentration of cancer cells is limited, and tissue irregularities are negligible as well as the volumes of tissues interested by the disease; therefore, hypoxia effect can be disregarded, and cancer cell dynamics can be considered as a perturbative effect on the normal dynamics or on the homeostasis of the compartments. Under these constraints, also a self-seeding phenomenon causing a feedback signal from the bone niche toward the breast lobular duct is negligible.

In our model, we also address the case of cancer progression when medical treatments are provided. More precisely, we focus our attention on the effects of bisphosphonates on cancer cells. Drugs represent a further form of coupling between the model compartments affecting the dynamics of the microenvironment and the cancer cells fitness landscapes (see [33, 34] for a description of fitness landscapes).

Under the assumption that the mean field approximation holds true (i.e. average over all the cell populations), the system dynamics is described in terms of ordinary differential equations (ODE) for molecule concentrations and cell population densities. Below, we present and discuss the system of ODEs for each compartment, we show the results obtained by numerical simulations and how we used the model in order to simulate different trajectories representing the disease evolution so to produce the respective survival probability curves.

**Branching processes and heterogeneity.** The possibility that cancer cells, developed in the breast, form metastasis in the bone tissue is due to the occurrence of driver mutations causing overexpression of specific proteins which help the cells to accomplish such process. The numerosity of these populations with improved pro-metastatic behaviour depends on their capability of surviving in a given environment. Indeed, the development of a mutation occurs during asymmetric proliferation, a rare process in which a cell divides in two daughter cells where one of the two is equal to the parent, while the other presents a mutation.

In our model, we focus on the number of cells that develop one given mutation or present simultaneously all the pro-metastatic mutations. Such multi-mutation path can be obtained through several *paths*, where a path is a sequence of mutations leading from the initial profile **j** = (0, 0, …, 0) (no mutation) to the final profile **j′** = (1, 1, …, 1) (all the genes in the path are mutated).

When the considered system is characterized by various cell groups, each of which is different from another due to specific properties, the branching process is the process used to dynamically link these cells together, as well as, to describe the relation between groups in terms of parents and offspring. As a consequence, in the present case, the acquiring of new genetic mutations by cancer cells can be described in terms of a branching process [13]. Let *x*_**j**_(*t*) be the expected number of cells at time *t* with **j** = (*j*_1_, *j*_2_, …, *j*_*m*_) driver mutations. Each component *j*_*i*_ of **j**, corresponding to the specific driver mutation *i*, can have value 1 if the mutation occurred, or zero otherwise. The length *m* of **j** is the minimum number of driver mutations necessary to perform an action (i.e. forming metastasis in the bone). We assume that each driver mutation with integer index *i* ∈ [1, *m*] can be caused by the variation of the state of a single gene.

A mutational path, 𝓟 = **j** → **j′**, corresponds to an ordered set of driver mutations **s** = (*i*_1_, …, *i*_*k*_, …, *i*_*m*_). We can choose another path by rearranging the driver mutations in a different order. The path 𝓟 describes the passage from the cell population *x*_**j**_, where *j*_*i*_ = 0 for all the elements *i* ∈ **s**, to the population *x*_**j′**_, where *j*_*i*_ = 1 for all the elements *i* ∈ **s**, through the sequence of acquired mutational steps: *i*_1_, …, *i*_*k*_, … *i*_*m*_.

If we consider only the cases in which each driver mutation gives the cells a single specific pro-metastatic capability (i.e. overexpression of a protein), and we also neglect the possibility for a cell and its progenies to loose such a capability due to future random mutations, then, for a specific path 𝓟, the evolution of the sub-population *x*_*k*_(*t*) of CTCs being at the *k*-th mutational step is given by:

$$\begin{array}{ccc} \partial_{t}x_{k}\left(t\right) & = & \overset{\mathrm{symmetric}\;\mathrm{proliferation}}{\overset{︷}{r_{b}\;\left(1-u_{0}\right)\;x_{k}\left(t\right)}}+(\overset{k-\mathrm{th}\;\mathrm{mutation}}{\overset{︷}{r_{b}\;u_{0}\;C_{k}\;x_{k-1}\left(t\right)}}-\overset{(k+1)-\mathrm{th}\;\mathrm{mutation}}{\overset{︷}{r_{b}\;u_{0}\;C_{k+1}\;x_{k}\left(t\right)}})-\overset{\mathrm{apoptosis}}{\overset{︷}{r_{d}\;x_{k}\left(t\right)}}, \end{array}$$ (1)

where *r*_*b*_ is the cell proliferation rate, *r*_*d*_ is the cell death rate, *u*_0_ is the punctual probability of mutation per unit of RNA expression level, *C*_*k*_ is the *k*-th gene expression level and *u*_0_ = *u*_*m*+1_ = 0. In Eq (1), the integer index *k* ∈ [0, *m*], and the initial conditions are *x*_0_(0) = 1 and *x*_*l*_(0) = 0 for any *l* ≠ 0.

In the RHS of Eq (1), the first term takes into account the proliferation of cells when none of the *m* driver mutations occur. The second term in the parenthesis describes the asymmetric proliferation of both the sub-populations *x*_*k*−1_ and *x*_*k*_ involved in *k*-th and (*k*+1)-th driver mutation of the path 𝓟, respectively. The last term represents the apoptotic process.

It is important to notice that on the one hand, when a cell mutates, it branches, and on the other hand, heterogeneity in the gene expression of a cell sub-population influences the probability of branching, or more precisely, it affects the time rate at which similar cells mutate. In order to take into account the genetic cell heterogeneity, we could have introduced a second index in the cell sub-population so to discriminate them in sub-populations of sub-populations. Nevertheless, due to the lack of specific datasets (at least to our knowledge) for gene expression on single CTCs derived from breast cancer, it would be difficult to determine the corresponding probabilities of mutation.

In this work, we focus on the tumour cells characterised by CD44, CD47 and MET mutations [5]. Hence, we apply Eq (1) to describe all the possible paths leading from the initial profile (0, 0, 0) to the final profile (1, 1, 1) with all the three genes mutated. By solving the corresponding system of equations, the number of cells with a profile **j^⋆^** corresponding to a single mutation at the *j*_*i*_-th position is given by:

$$\begin{array}{ccc} x_{j^{⋆}\left(t\right)} & = & x_{0}\left(0\right)\;e^{r\;t}\left(1-e^{-r_{b}\;u_{0}\;C_{i}\;t}\right), \end{array}$$

where *r* = *r*_*b*_−*r*_*d*_.

It is very convenient to rewrite the solutions independently from the specific traversed path and order of mutations in terms of sub-populations $\bar{x_{𝓓}}(t)=\sum_{\left\{j|j_{k}=1\forall k\in 𝓓\right\}}x_{j}(t)$ of cells which have acquired at least a specific sub-group of pro-metastatic behaviours 𝓓. Rearranging the solution of Eq (1), we have:

$$\begin{array}{c} \bar{x_{𝓓}}\left(t\right)=\bar{x_{0}}\left(t\right)\prod_{k\in 𝓓}[1-e^{-r_{b}\;u_{0}\;C_{k}\;t}], \end{array}$$ (2)

where $\bar{x_{0}}(t)=x_{0}(t)=x_{0}(0)\;e^{r\;t}$ is the sum of all the sub-populations (see Supplementary Information S1 Text for the mathematical derivation). From Eq (2), the ratio $\frac{\bar{x_{𝓓}}(t)}{\bar{x_{0}}(t)}$ is a number in [0, 1] representing the portion of cells with 𝓓 mutations. Identifying this ratio with the joint probability of a cell having those pro-metastatic properties derived by the 𝓓 mutations and under the condition that each driver-mutation occurs independently from the others, it follows that $\frac{\bar{x_{𝓓}}(t)}{\bar{x_{0}}(t)}=\prod_{k\in 𝓓}\Gamma (C_{k})$, where each Γ(*C*_*k*_) corresponds to the probability of acquiring the pro-metastatic behaviour *k*.

In order to describe the effects of EPCAM on CTCs, we consider a first part of the branching process, strictly related to EPCAM, which occurs on breast cancer cells and identifies the cells that can intravasate.

Considering all the tumour cells that are about to enter the near blood vessels, they will have a small probability of proliferating as CTCs; hence, all their pro-metastatic behaviours are due to previous cell divisions and mutations. Consequently, the second part of the branching process (related to CD47, CD44 and MET) occurs while tumour cells are still in the mammary duct.

In the blood vessels, cells with low values of CD47 are attacked by the immune system and eliminated; therefore, only cells with sufficiently high CD47 proteins on their surfaces can evade the immune system. More precisely, the higher the concentration of CD47, the longer the survival probability of CTCs is. The proteins CD44 and MET are involved in the extravasation process of circulating cells. Hence, their absence contributes to the permanence of the tumour cells in the circulatory system, and their presence contributes to characterise the component of CTCs population able to reach the bone and seed.

The branching process strictly divides the CTCs population in sub-populations of circulating cells labelled by specific driver mutations which follow specific cell behaviours. Nevertheless, in the blood stream CTCs follow trajectories which are much less distinct. The causes are due to the interactions with the microenvironment which are responsible for the selection on the basis of the four proteins concentrations and give rise to variability and further heterogeneity.

Based on the results in [5], we consider only the four proteins overexpressed in CTCs with a high potential of generating bone metastasis: EPCAM, CD44, CD47 and MET. Nevertheless, the method can be extended, or modified to include other proteins for other type of cancers. In Simulations subsection, we discuss what happens increasing or decreasing the minimum number of proteins necessary for creating metastasis.

**Mammary duct compartment.** In order to describe the tissue dynamics as populations of healthy and mutated cells, we introduce a branching process based on the tissue scale model proposed in [2] where the cell populations are *ρ*(*ϕ*, *t*) and the index *ϕ* ∈ [0, Φ] represents the cell state which is identified with the cell phenotype.

We perform an order parameters reduction of that model neglecting the intra/extra-cell scale equations since the reactions involved are much faster than those at the tissue level. Hence, the TGF-*β* synthesised, activated and bounded with the receptors on the cells membrane $R_{\text{ec}}^{⋆}(\phi )$, which are internalised so to generate the signalling inside the epithelial cells of the mammary duct, can be considered constant without significantly affecting the dynamics at larger scales. The TGF-*β* values are set equal to those at the equilibrium reached during the dynamical evolution of the tissue sub-system. We neglect also asymmetric proliferation and we constrain the cells to change their phenotype only in sequential steps.

Using the same terminology in [2], healthy cells have phenotype *ϕ* = 0, pre-neoplastic cells are indexed as *ϕ* = 1, tumoural cells correspond to *ϕ* = 2 and cells with aggressive tumoural behaviour and strong resistance to TGF-*β* inhibiting signalling have phenotype *ϕ* = Φ = 3. We associate the cell phenotype to the TGF-*β* which is one of the proteins involved in the reduction of cell-to-cell E-cad connection. Hence, the activated TGF-*β*, when internalized, induces morphological changes on the cells which become more round and unconstrained. The TGF-*β* synthesised by cancer cells with index *ϕ* > 0 are more elevated than the quantity produced by healthy cells; therefore, the higher is the index *ϕ*, the higher is the chance it moves and/or positions itself unrespective of the morphological structure of the tissue. It is important to remark that the index *ϕ* is not related to the expression of proteins involved in the metastasis formation processes.

The equation governing the cell sub-populations density *ρ* of the mammary duct epithelium tissue in a unit volume containing a cell and its nearest neighbour cells at time *t* and having phenotype *ϕ* is:

∂tρ(ϕ,t)=rp(1−δϕ,0ρ0˜Cϕ−∑0≤η≤Φη≠ϕρ(η,t)Cη)(1−∑0≤η≤Φρ(η,t)Cη)ρ(ϕ,t)Rec⋆(ϕ)g(ϕ)︷symmetriccellproliferation+−∑ϕ=0ϕρ(ϕ,t)CϕraRec⋆(ϕ)g(ϕ)ρ(ϕ,t)︷TGF−βinducedapoptosis+∑ϕ=0ϕρ(ϕ,t)Cϕrm[(1−δϕ,0)ρ(ϕ−1,t)−(1−δϕ,Φ)ρ(ϕ,t)]︷cellmutation∑212+−∑ϕ=1ϕ−1ρ(ϕ,t)Cϕrint(1−δϕ,0)ρ(ϕ,t)Γ(CEPC)︷cellsenteringthebloodstream∑212. (3)

The first term on the RHS of Eq (3) represents the proliferation process of cells. The factors in the parentheses take into account the maximum volumetric capacity *C*_*ϕ*_ and the minimum capacity $\tilde{\rho_{0}}$ left to healthy cells by cell populations with *ϕ* > 0, respectively. Cell proliferation is regulated by the TGF-*β* entering the cell ($R_{\text{ec}}^{⋆}$), and the effect of this protein depends on the phenotype sensing exponent *g*(*ϕ*). For non-tumoral cells (*ϕ* < 2), the capacity *C*_*ϕ*_ expresses the average maximum number which can lay on the surface of the mammary duct, and for tumoral cells (*ϕ* ≥ 2), it represents the average maximum number of cells which can be hosted above, below and on the surface of the mammary duct. The second term describes the apoptosis induced by the TGF-*β* and depends on the phenotype sensing exponent *g*(*ϕ*). For sub-population with phenotypes *ϕ* < Φ, the exponent *g*(*ϕ*) are non-negative and decreasing with *ϕ*; consequently, higher quantity of TGF-*β* inhibits proliferation and increases the apopotosis rate of these sub-populations. On the contrary, *g* is negative when *ϕ* = Φ. Therefore, TGF-*β* enhances the proliferation and reduces the apoptosis of the aggressive population highlighting the anti-oncogenic and pro-oncogenic role of TGF-*β* on different cell populations. The third terms expresses the mutation transition of a cell from a state *ϕ* to the state *ϕ*+1, and the delta of Kronecker *δ*_*α*,*β*_, which is 1 for *α* = *β* and 0 otherwise, implies that there are no cell which mutate to healthy cells and no further mutation occurs on cells in the state Φ.

The last term on the RHS of Eq (3) is the first step of the branching process relative to the expression of cell membrane proteins favouring the formation of metastasis, and it describes the intravasation of cancer cells in the nearest blood vessels occurring at rate *r*_*int*_ with probability Γ(*C*_*EPC*_), where *C*_*EPC*_ indicates the EPCAM gene expression level (see Methods section and Branching process and survival probability prediction subsection). Overexpression of EPCAM increases the probability that a cell per unit of time passes through the nearest cells and reaches the circulatory system. Hence, because of driver mutations and over-production of TGF-*β*, cancer cells with EPCAM overexpression will easily unbind from the neighbour cells increasing their chance of reaching the local blood vessels and becoming CTCs; therefore, only cells with *ϕ* > 0 contributes in generating CTCs.

It is worthy to notice that there is an obvious relationship between the cell density phenotypes *ρ*_*ϕ*_ and the frequencies of the branching process populations *x*_*k*_. The index *ϕ* refers to mutations inducing TGF-*β* resistance, and the index *k* refers to mutations affecting the expression of the three specific markers on the membrane of bone metastasising cancer cells. The former mutations are related to the behaviours of the source of the CTCs (the epithelial cells in the breast), while the latter are related to the behaviours of the CTCs. Hence, mutations altering the normal TGF-*β* signalling will propagate their effects on the concentration of the populations *x*_*k*_. Furthermore, in a complex biological process as the breast cancer cells metastasising in the bone, the order and the times at which all these mutations occur might play a relevant role. Nevertheless, for the sake of simplicity, we divided the two type of mutations (indexed *ϕ* and *k*, respectively) into two independent groups. Therefore, the two types of mutations occur in parallel introducing only a partial complexity in the system and disregarding further time interdependent causalities.

**CTCs in the bloodstream compartment.** After the first step of the branching process depending on the expression of EPCAM, the remaning branching of cancer cells discriminates groups of CTC sub-populations with different genetic characteristics. All the sub-populations of cancer cells with overexpressed EPCAM, by definition, will intravasate, but not all of them will survive to the immune system control and not all of them will be able to extravasate and seed. The outcomes and the time of permanence of CTCs in the blood system depends on the properties of the CTCs themselves. Indeed, only a small component of all the CTCs with sufficient high pro-metastatic behaviours have high chances in forming metastasis. For example, CTCs with low CD47 are eliminated by the immune system control at rate *r*_*imm*_, and even though they might have high CD44 or MET, they will have a small chance of surviving and a short lapse of time to attempt extravasation. Similarly, cells with high CD47 will have a high chance of surviving the immune system attacks, but if they express low quantity of CD44 or MET proteins, they do not have a high probability of forming metastases. Nevertheless, since these CTCs remain longer in the circulatory system, they can attempt to extravasate more times with rate *r*_*ext*_. The time evolution of the CTCs population is described by the following equation:

∂tCTC(t)=rintVROI∑ϕ=1Φρ(ϕ,t)Γ(CEPC)︷intravasatingCTCs−∑a=1Φ1rimmCTC(t)Γ(C47¯−C47)︷CTCsdonotescapetheimmunesystem+−∑aaarextCTC(t)Γ(C44)Γ(C47)Γ(CMET)︷extravasatingCTCs. (4)

and the equation for the extravasating CTCs, *CTC*_*e*_, is:

∂tCTCe(t)=∑aaarextCTC(t)Γ(C44)Γ(C47)Γ(CMET)︷extravasatingCTCs−∑aaarseedCTCe(t)︷seedingCTCs. (5)

The first term on the RHS of Eq (4) represents the outward flux of epithelial cells escaping from the mammary duct into the bloodstream. The parameter *V*_*ROI*_ is used to relate the amount of CTC produced in the breast unit of volume, which encloses a cell and the neighbour ones, with the unit of volume of blood. We suppose that healthy cells (*ϕ* = 0) do not generate CTCs, and the total amount of epithelial cells entering the blood stream *V*_*ROI*_ *r*_*int*_ Γ(*C*_*EPC*_) increases with the synthesis of EPCAM, where Γ(*C*_*EPC*_) is the probability of having EPCAM overexpressed (see Methods section and Branching process and survival probability prediction subsection). Moreover, by increasing the value of *r*_*int*_, it would be possible to describe the flux of clusters of CTCs presented in [21]. The second term in Eq (4) describes the CTCs incapacity of surviving against the attacks of the immune system. Therefore, it expresses the amount of CTCs eliminated by the immune defense. The probability that a cell does not survive depends on the expression level of CD47. Indicating with $\bar{C_{47}}$ the maximum value of CD47 overexpression (see Methods section), higher is the difference $\bar{C_{47}}-C_{47}$, lower is the probability of escaping the immune system attack. The third term deals with the effects of CD44, CD47 and MET, which are the genes contributing to the invasiveness of CTCs and to the increased capability of CTCs in generating metastasis (*C*_44_, *C*_47_ and *C*_*MET*_ correspond to the gene expression levels of CD44, CD47 and MET respectively, see Methods section and Branching process and survival probability prediction subsection). Hence, the third term is the flux of CTCs exiting the bloodstream, and it appears with opposite sign on the RHS of Eq (5). The second term in Eq (5) represents the cell seeding in the bone tissue at rate *r*_*seed*_.

### Bisphosphonates administration to metastatic breast cancer patients

Activation and maturation of osteoclasts during bone repairing are highly reduced by bisphosphonates which strongly bind to hydroxyapatite and prevalently impregnates sites characterised by elevated bone remodelling activity [2]. Various works have confirmed the important role of bisphosphonates on cell types not directly involved with the osteoclastogenesis, but which are related to metastasis formations in the bone tissue highlighting the antioncogenic effects of the drug. Clinical trials have shown that breast cancer patients (with or without bone metastasis) increase their disease free survival when treated with bisphosponates [14]. Recently, studies on breast cancer have shed light on some of the underlying mechanisms of the interaction between bisphosphonates and different breast cancer cell lines [35–37]. The epithelial-mesenchymal transition (EMT) activated by the TGF-*β* induces the epithelial cells of the lobular duct to undergo cellular changes which cause the reduction of cell-to-cell contacts, the loss of cell polarity and the weakening of the cytoskeletrical structure [38]. The resulting effects are single cells detached from the surrounding tissue forming elongated laminopodia-like or filopodia-like structures, with increased capability of migrating and proliferating [39, 40].

Cancer cells treated with bisphosphonates reduce the formation of protrusions, and promotes the expression of epithelial markers to the detriment of mesenchymal markers. In fact, bisphosphonates revert the EMT before cancer cells unbind from the nearest cells reducing the aggressiveness of unbound cells. In benign cancer breast tissue, epithelial cells in the lumen express low levels of the epithelial marker EPCAM, but in different tumour tissues, including breast cancer, cell adhesion molecules are overexpressed. This is in accordance with the statement in [15] that reversing of the EMT occurs prior to the detachment of luminal cells, and it is in agreement with the experiment in [5]. Bisphosphonates have two other major direct effects on cancer cells. First, they slow down the proliferation rate of breast cancer cells by arresting both the G1 and the S phase of the cell cycle [15] [41]. Second, the treatment is also responsible for increasing the apoptosis signalling of epithelial cells [37]. As reported by the authors in [42], the apoptotic signalling induced by bisphosphonates is more enunciated in aggressive breast cancer cells while this effect is mitigated in low and non-tumourigenic cells; hence, this differentiated tumoural action causes bisphosphonates to contrast the effects due to overproduction of TGF-*β* and, at the same time, it supports the suitability of the bisphosphonates for a pharmacological intervention on patients with breast cancer [14]. The use of bisphosphonates in breast cancer therapy has been the target of intensive studies that have elucidated several aspects of the interaction between the drugs, cancer subtypes and pathways ([14], [15], [35], [36], [37], [43], [44], [45], [46]). International clinical trials have found a striking differences of bisphosphonates treatment effects between pre-menopausal and post-menopausal women (see [42], [47], [48], [49], [50], [51] among others; Ingunn Holen (Sheffield), Daniele Parenti (Parma), Ignacio Tusquets Trias de Bes (Barcelona) and Andreas Trumpp (Heidelberg) personal communications).

The time of permanence of CTCs in the circulatory system depends on their own characteristics allowing them to survive or extravasate. Indeed, while in the blood vessels, cancer cells are attacked by the immune system. In this environment, the TGF-*β* released by the tumoural cells activate the immune surveillance and help immune cells to detect the CTCs. At the same time, TGF-*β* signalling modulates platelet aggregation around the CTCs [52] [53]. Platelet aggregations shield CTCs and protect them against immune mediated clearance [54]. Furthermore, platelets create a micro-environment favouring the EMT of CTCs [53] [55].

It has been shown that high concentration of bisphosphonates in the circulatory system, for example by intravenous administration, strongly affect the survival of CTCs. Bisphosphonates reduce the activation and aggregation of platelets [56] [57], and diminish the probability of platelets of both preferentially intercepting CTCs and fostering agglomeration around CTCs. Therefore, bishosphonates make CTCs more susceptible to immune cell attacks and direct bisphoshonates apoptotic signalling.

**Bisphophonates: treatment in the compartments.** Bisphosphonates as zoledronate and alendronate have been adopted against osteoporotic diseases due to the inhibitory interaction of the drug against the mature osteoclasts in the resorption of the bone mineral matrix. Only recently, bisphosphonates have been used as an anti-cancer drug. In the work [14], the authors studied the effects of zoledronic acid on a cohort of 33 patients with breast cancer and on a cohort of 20 patients with breast cancer and bone metastasis. The patients were treated by intravenous administration of 4 mg of bisphosphonate diluted in 100 ml of saline solution to study the variations of the CTCs during the following 14 days. In order to describe the time evolution of the concentration of bisphosphonate per 7.5 ml of blood (*BP*), we introduce the following equation:

∂tBP(t)=∑aaaH(t−t1)H(t2−t)BP¯t2−t1︷drugadministration−∑aaadBPBP(t)︷drugdecay+−∑aaarBPBP(t)[σ0∑ϕ=1Φρ(ϕ,t)+CTC(t)+CTCe(t)]︷drugabsorption. (6)

where $\bar{BP}$ is the total amount of bisphosphonate administered, [*t*_1_, *t*_2_] is the dispense time interval and *H*(*t*) is the heaviside step function. The first term expresses the drug administration while the second one describes the drug decay at rate *d*_*BP*_. The last term takes into account the absorption of the drugs in all the compartments (mammary duct—$\sum_{\phi =1}^{\Phi }\rho (\phi ,t)$, CTCs and bone—*CTC*_*e*_(*t*)). The terms of interaction do not present any time delay between the concentration of the drug and the population of cancer cells in each compartment. The reason is that the intravenous bisphosphonate rapidly diffuses in the circulatory system distributing homogeneously all over the systems while slowly relaxes toward the equilibrium; therefore, the drug reaches the compartments approximatively at the same time, and the concentration will remain equal among the compartments at any time.

The interaction introduced by the bisphosphonates represents a further form of coupling between the compartments; indeed, if we could exclude one of the compartments, we would have less dispersion of bisphosphonates and an increase of drug absorbed by the remaining compartments.

In order to reproduce the effects of the bisphosphonates on the CTCs in the blood system, in the ductal lobular unit (DLU) and in the bone multicellular unit (BMU), we added the interaction terms between the administered bisphosphonates and the cancer cells in each compartment. These terms of interactions work as sink of cancer cells and they are linear in the concentration of the drug and the interacting cells. Therefore, we modified the ODEs system Eqs (3–5) by adding the following terms describing the therapy effect on the right hand side of:

- the equation for the cell populations in the mammary duct, Eq (3)
  $$\begin{array}{c} -r_{BP}\;BP\left(t\right)\;\rho (\phi ,t), \end{array}$$ (7)
- the equation for the *CTC* in the circulatory system, Eq (4)
  $$\begin{array}{c} -r_{BP}\;BP\left(t\right)\;CTC(t), \end{array}$$ (8)
- the equation for the *CTC*_*e*_ in the bone niche, Eq (5)
  $$\begin{array}{c} -r_{BP}\;BP\left(t\right)\;CTC_{e}\left(t\right) \end{array}$$ (9)

For the full set of model equations see Supporting Information S2 Text.

In [14], the two cohort of patients (immediately after they were diagnosed with breast cancer and matched the baseline characteristics for the study) were treated with bisphosphonates and observed during the biological window of 14 days in which no chemotherapy, radiotherapy or other types of drugs were administered. During this period, the absence of further treatments highlighted the effect of bisphosphonates on the patients. The counting of CTCs in the blood during the biological window provided us with suitable measurements for tuning some parameters of our model in order to reproduce the CTCs enumeration. It is important to stress that we address the early stages of breast cancer; therefore, we describe cases which have the initial conditions corresponding to a healthy state, and we run the system until the cancer in the breast reaches a detectable size. Comparing the cases with and without bisphosphonates, in the former case, CTCs increase monotonically; while in the latter, the curve of CTCs abruptly decreases immediately after the drug administration (see Simulations subsection). We used the model in order to identify the regions of time when the administration of bisphosphonates has the most effect. From our simulations, we have seen there is a specific time before which it holds true that the sooner the bisphosphonates are injected, the more the number of CTCs decreases. However, the time at which breast cancer is diagnosed represents the minimum time limiting when the bisphosphonates can be administered.

In our multi-compartment model, the treatment allows us to investigate the response of the systems to the external perturbation. In the next subsections, we discuss how the drug permits us to investigate the connections between the cancer cell concentrations in the compartments and the survival probability of patients, as well as, the effects of further necessary mutations in the branching process and the delay in the immune system response.

### Branching process and survival probability prediction

We consider two types of survival probabilities: the overall survival and the progression free (PF) survival. The former is the probability curve given by the measurement of times which goes from the moment of diagnosis of the disease to the occurrence of the event “death”. The latter is the curve describing the probability given by the measurement of times which goes from the administration of the treatment to the worsening of the disease.

In order to create a survival distribution, we define two groups of parameters: the fixed parameters which describe the baseline characteristics common to all the elements in the sample set (patients in the cohort) and the stratification parameters identifying the properties of specific sub-groups of patients. The fixed parameters are set to the same constant values in all the simulations, while the stratification parameters are chosen randomly for each simulated patients.

The time of the event “death” used in generating the overall survival is defined as the first occurrence time of the following relation: *ρ*(2, *t*)+*ρ*(3, *t*)+*σ*_1_ *CTC*(*t*) > *ρ*(0, *t*)+*ρ*(1, *t*), which compares the sum of the most aggressive cell phenotypes (*ρ*(2, *t*) and *ρ*(3, *t*)) and CTCs with the sum of the populations of healthy and pre-neoplastic cells (*ρ*(0, *t*) and *ρ*(1, *t*) respectively). The event of change of patient’s condition identifying the disease worsening in the progression free survival is set as the first occurrence time at which $CTC_{e}>\bar{CTC_{e}}$ holds true, where $\bar{CTC_{e}}$ is the mean value of extravasating CTCs over all the sample, see Methods section, Tables 1 and 2.

**Table 1:** List of variables.

TGF-*β* receptor-ligand	$R_{\text{ec}}^{⋆}(\phi )$	{14.625, 28.364, 19.847, 16.888}
number of nearest neighbour cells with phenotype *ϕ*	*ρ*(*ϕ*, *t*)	$\left\{6,0,0,0\}\;[{V_{\text{neigh}}}^{-1}\right]$
Circulating tumour cells	*CTC*	$∼0\;[{V_{\text{blood}}}^{-1}]$
extravasating tumour cells	*CTC*_*e*_	$∼0\;[{V_{\text{blood}}}^{-1}]$
Bisphosphonates concentration	*BP*	0 [mg ml^−1^]

If the variable depends on the phenotype *ϕ*, then we give a list of values sorted by increasing order of the phenotype. References and choices for the numerical values of the initial conditions are discussed in the Results section.

**Table 2:** List of parameters.

max phenotype index	Φ	3
proliferation rate	*r*_p_	1.1 × 10^−6^ [s^−1^]
apoptosis rate	*r*_a_	9.89 × 10^−8^ [s^−1^]
mutation rate	*r*_m_	1.1 × 10^−11^ [s^−1^]
proliferation exponent	*g*_p_(*ϕ*)	{0.12, 0.06, 0.001, −0.06}
apoptosis exponent	*g*_a_(*ϕ*)	{0.12, 0.06, 0.001, −0.06}
cell capacities	*C*_*ϕ*_	$\left\{6,6,8,8\}\;[{V_{\text{neigh}}}^{-1}\right]$
intravasation rate	*r*_*int*_	2.5 × 10^−6^ [s^−1^]
pre-CTC proliferation rate	*r*_*b*_	5.9 × 10^−8^ [s^−1^]
pre-CTC death rate	*r*_*d*_	5.26 × 10^−8^ [s^−1^]
point mutation rate	*u*_0_	5. × 10^−8^ [s^−1^]
immune response rate	*r*_*imm*_	10^−7^ [s^−1^]
extravasation rate	*r*_*ext*_	5.6 × 10^9^ [s^−1^]
EPCAM expression level	*C*_*EPC*_	1.0828
CD47 expression level	*C*_47_	1.0363
CD47 alert threshold	$\bar{C_{47}}$	3.2976
CD44 expression level	*C*_44_	1.0971
MET expression level	*C*_*MET*_	1.0713
seeding rate	*r*_*seed*_	1.1 × 10^−8^ [s^−1^]
administered bisphosphonates	$\bar{BP}$	4 [mg ml^−1^]
drug interaction rate	*r*_*BP*_	10^−3^ [*V*_blood_ s^−1^]
drug decay rate	*d*_*BP*_	2⋅10^−3^ [s^−1^]
dimensional constant	*σ*_0_	$30\;[V_{\text{neigh}}\;{V_{\text{blood}}}^{-1}]$
dimensional constant	*σ*_1_	$1\;[V_{\text{blood}}\;{V_{\text{neigh}}}^{-1}]$

If the parameter depends on the phenotype *ϕ*, then we give a list of values sorted by increasing order of the phenotype.

All the times defined above are larger than the time *t* ^⋆^ when the tumour reaches a detectable size which for medical and gene expression data corresponds to the time when patients are diagnosed with breast cancer. In the simulated trajectories, the time of detection corresponds to *t* ^⋆^ ∼ 2 years of evolution of the system starting from a healthy initial condition and developing breast cancer.

**Fig 2:**
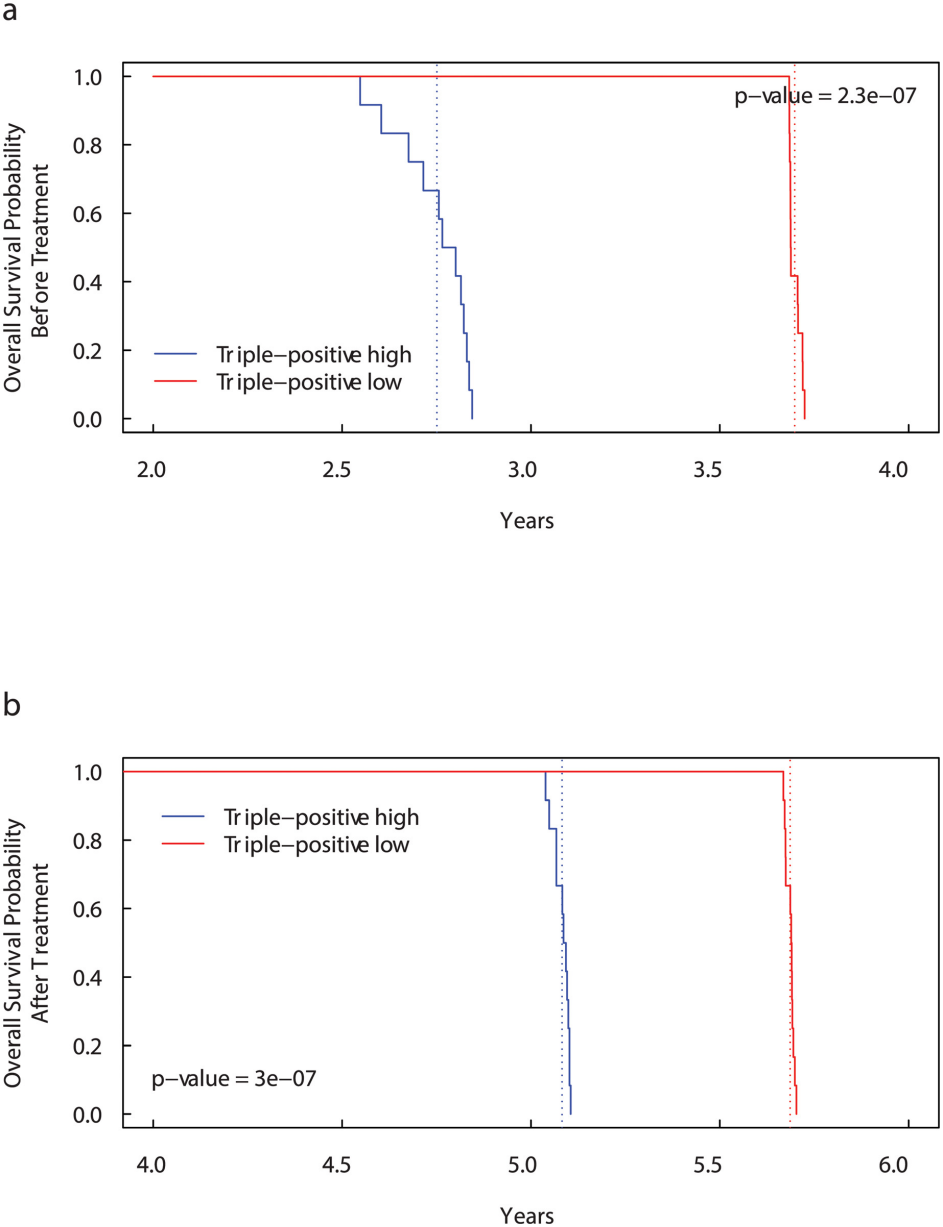
Kaplan-Meier curves for Overall Survival Probability on CD44, CD47 and MET expression groups before (a) and after (b) bisphosphonates administration. Patients with more than the median number of *CD*44^+^ *CD*47^+^ *MET* ^+^ cells (“Triple-positive high” patients) have significantly lower overall survival than “Triple-positive low” patients [58]. The dotted lines show the median overall survival times. Before treatment: 2.75 years for “Triple-positive high” patients versus 3.70 years for “Triple-positive low” patients. After treatment: 5.08 years for “Triple-positive high” patients versus 5.69 years for “Triple-positive low” patients.

To highlight the role of the overexpression of the four proteins (EPCAM, CD47, CD44 and MET) in the development of breast cancer and understand the relationship between CTCs (with and without pro-metastatic behaviours) and survival times, we compare simulated overall survival probabilities in the case of CD44, CD47 and MET overexpressions versus the case in which the three CTCs’ markers are underexpressed. In the simulations, we focused only on CD44, CD47 and MET overexpressions and underexpressions (excluding EPCAM) because we are considering the cancer cells that are already in the blood vessels. Hence, we are assuming that in all these cells EPCAM is already overexpressed. In particular, we randomly generated a first group of 12 patients with overexpressed gene markers and a second group of 12 patients with underexpressed values of the proteins. We defined a marker overexpressed (underexpressed) if its gene expression level is higher (lower) than the average value (*C*_44_, *C*_47_ and *C*_*MET*_) extracted from the mRNA datasets, see Methods section and Table 1. Fig 2a shows that patients with all the three markers overexpressed (“Triple-positive high” patients [58]) have a survival probability lower than patients with underexpressed markers (“Triple-positive low” patients), in accordance with [5].

Adopting the same conditions used in the previous cases, in Fig 2b, we present the survival curves obtained with the administration of a single dose of 4 mg/100 ml of bisphosphonate for 15 minutes starting at time *t* ^⋆^. Even after the bisphosphonates administration, the “Triple-positive high” patients present a lower survival probability than “Triple-positive low” patients. However, including the drug administration the median overall survival times increase by about 2 years in both the cases (from 3.36 to 5.77 years in “Triple-positive high” patients and from 3.82 to 5.91 years in “Triple-positive low” patients).

**Fig 3:**
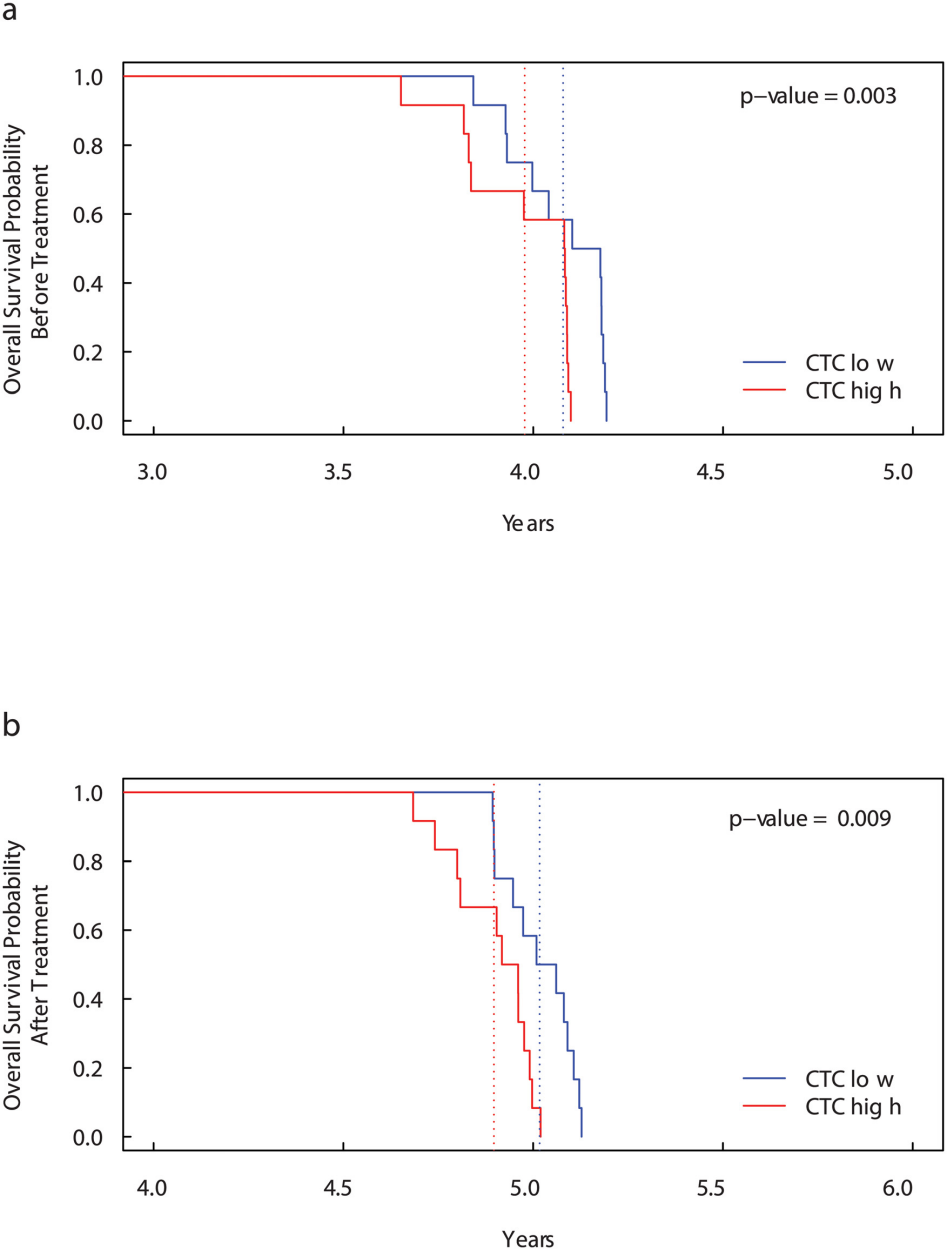
Kaplan-Meier curves for Overall Survival Probability on CTC level groups before (a) and after (b) bisphosphonates administration. Patients with more than 5 CTCs per 7.5 ml blood (“CTC high” patients) have significantly lower overall survival than “CTC low” patients. The dotted lines show the median overall survival times. Before treatment: 3.98 years for “Triple-positive high” patients versus 4.08 years for “Triple-positive low” patients. After treatment: 4.90 years for “Triple-positive high” patients versus 5.02 years for “Triple-positive low” patients. The high number of CTC cells is an indicator of decreased overall survival.

In order to analyse the effects of high concentration of CTCs in the blood, we compare the overall survival probability obtained from the 24 patients simulated in the previous case. However, in this case, we divided the 24 patients into two different groups by separating those which at time 1.7 × 10^8^ seconds (time at which the amount of CTCs are sufficiently elevated) have more than 5 CTCs per 7.5 ml of blood (“CTC high” patients) from those which have less or equal to 5 CTCs per 7.5 ml of blood (“CTC low” patients). We predict the overall survival probability without treatment in Fig 3a and with a single dose of bisphosphonates in Fig 3b. The method of selection does not guarantee that the group with high number of CTCs has also an high values of extravasating CTCs and vice versa. Indeed, in both the figures, the survival curves for the two groups are very close one another.

**Fig 4:**
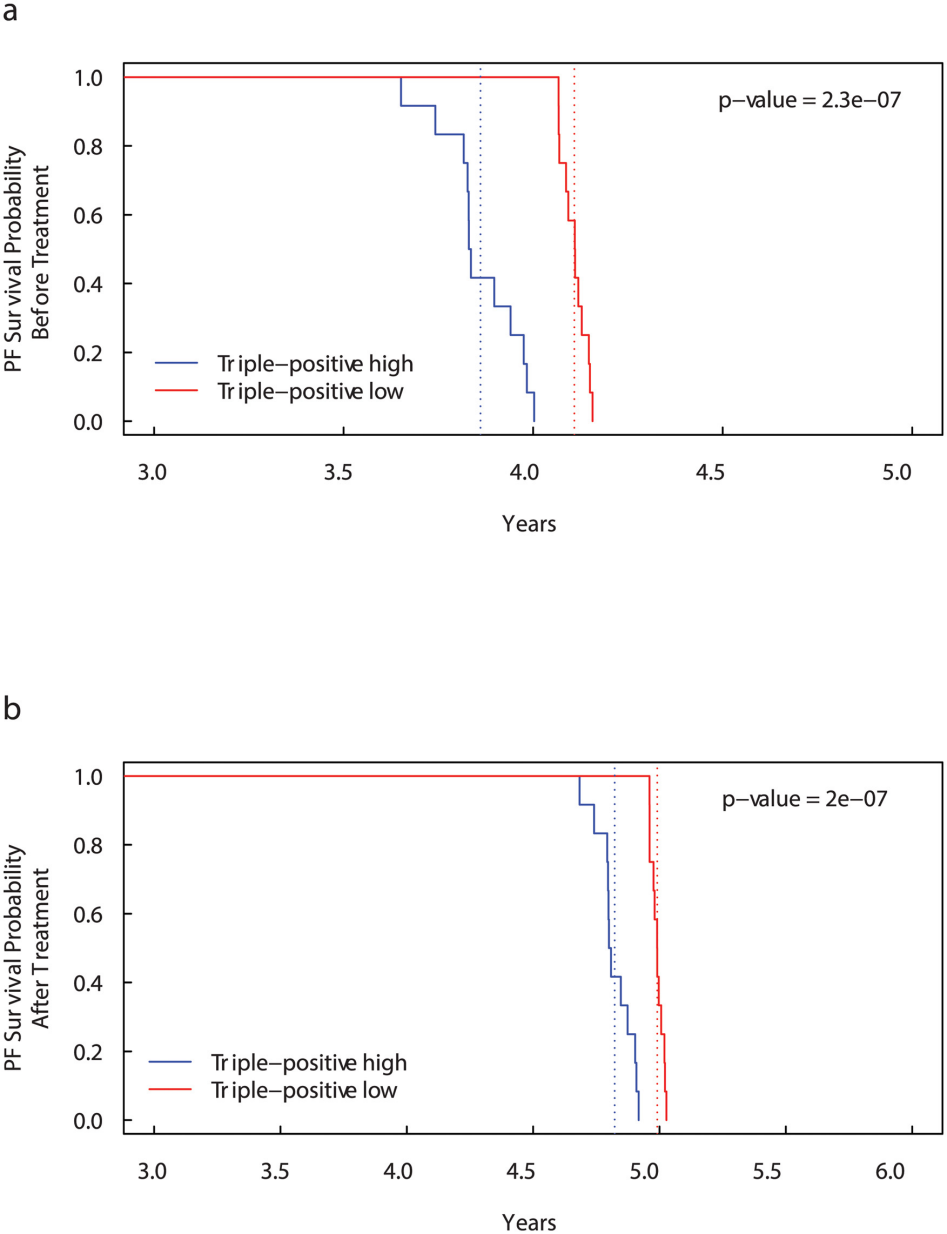
Kaplan-Meier curves for Progression-Free (PF) Survival Probability on CD44, CD47 and MET expression groups before (a) and after (b) bisphosphonates administration. Patients with more than the median number of *CD*44^+^ *CD*47^+^ *MET* ^+^ cells (“Triple-positive high” patients) have significantly lower overall survival than “Triple-positive low” patients. The dotted lines show the median overall survival times. Before treatment: 3.86 years for “Triple-positive high” patients versus 4.11 years for “Triple-positive low” patients. After treatment: 4.82 years for “Triple-positive high” patients versus 4.99 years for “Triple-positive low” patients.

In Fig 4a and 4b, we compare the PF survival curves with and without administration of bisphosphonates in “Triple-positive high” and “Triple-positive low” patients, simulated as in the first case. As before, patients with all the three markers overexpressed have median PF survival time lower than “Triple-positive low” patients (3.11 and 4.01 years respectively). After treatment, the median PF survival times increase in both the case reaching 5.34 years for “Triple-positive high” patients and 5.58 years for “Triple-positive low” patients.

**Fig 5:**
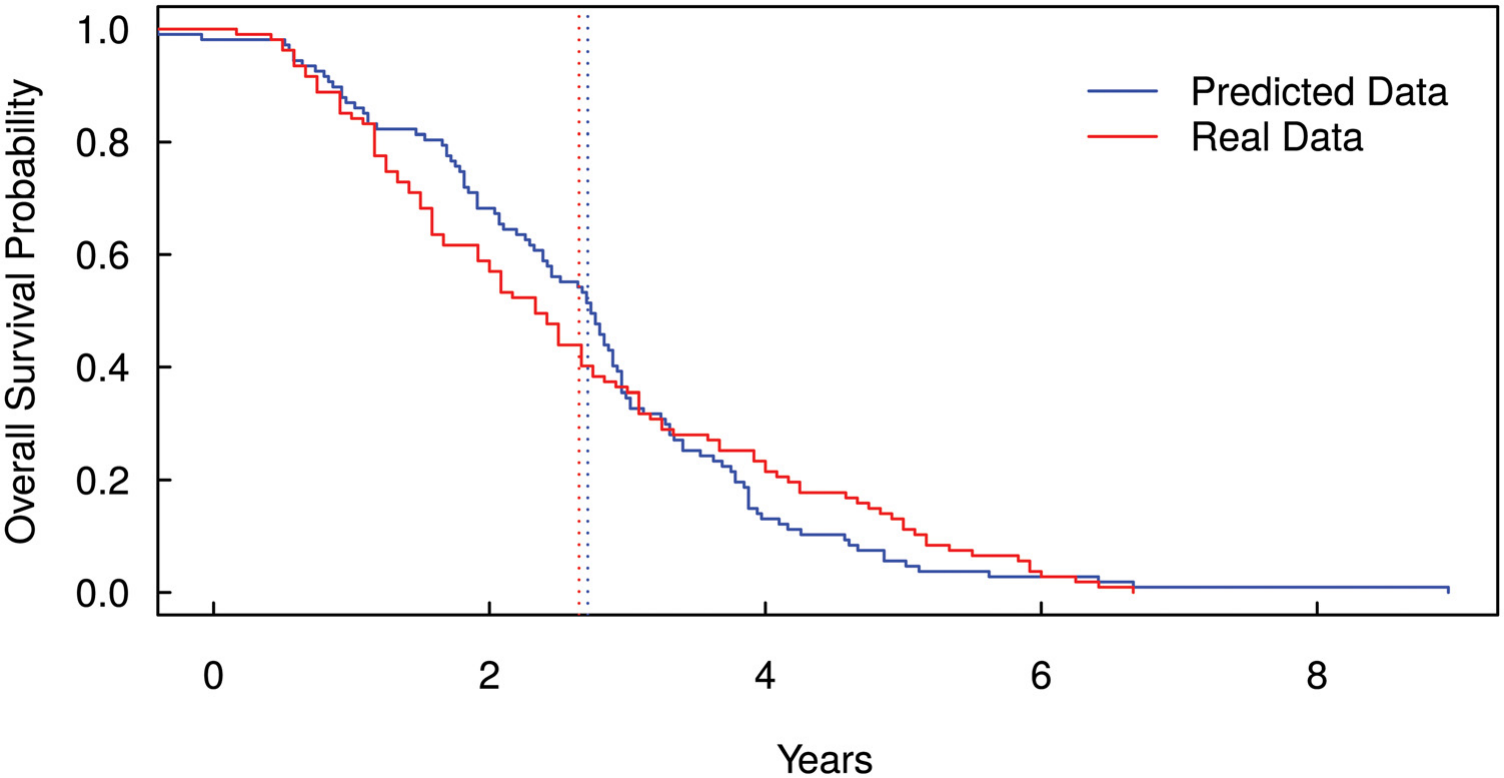
Kaplan-Meier curves for Overall Survival Probability Prediction. The figure shows the comparison between the real survival curve provided by a breast cancer dataset (accession number GSE2034) and the survival curve predicted by the model shifted back of 2 years. The shifting back was necessary because the model describes the time from the first driver mutation to the death, while the follow up period provided by the real data starts when the cancer has been diagnosed. The dotted lines show the median overall survival times (real data 2.65 years and predicted data 2.71 years).

In order to compare the survival prediction of the model with existing cancer data, we ran our model by using the data in GSE2034. The dataset provides gene-expression profiles and survival times for a cohort of 286 primary breast cancer patients with distant metastasis. By using the normalised gene-expression profiles of EPCAM, CD44, CD47 and MET, we computed the overall survival probability, as shown above, for each patient in the cohort. Fig 5 shows the comparison between the real survival curve provided by the analysed dataset and the survival curve predicted by the model. It is important to note that the predicted survival curve reported in Fig 5 has been shifted back of 2 years. The shifting was necessary because the starting time of the model corresponds to the first-mutation time, while the follow up period presented in the real data starts when the cancer has been diagnosed. Hence, the time computed by the model includes the time necessary to the tumour to reach a detectable size and to be diagnosed. Further analysis on the shifting-back time could be done to analyse the time when the first driver mutations took place.

### Simulations

Simulations consist in numerically solving with the explicit Runge-Kutta method the set of ODEs Eqs (3–5), which represent the general case, and the equations relative to the special cases Eqs (6–9). The initial conditions adopted in all the simulations represent an individual in healthy state. More precisely, this means that in the DLU which is going to develop the disease, all the cells are healthy epithelial cells with no driver mutations, *ρ*(0, 0) = 6 and *ρ*(*ϕ* > 0, 0) = 0 [2]; furthermore, the CTCs are zero and consequently also the extravasating CTCs are null, see Table 1.

In each case, the parameters are divided into fixed parameters and stratifying parameters. The former are set constant and are derived from the literature, while the latter are drawn from the distributions derived from mRNA datasets, or they are chosen equal to the average values depending on the type of simulation. All the averaged parameter values are listed in Table 2. The Sensitivity Analysis of the parameters is shown in S3 Text and S1–S12 Figs.

Assuming tumours of spherical shapes, we set the ductal carcinoma detectable size equal to a volume with an average diameter of 6 mm corresponding to 35% mammography screening test sensitivity [59]. The time *t* ^⋆^ at which the tumour (simulated with nominal values of the parameters, Table 2) reaches the detectable size is 7 × 10^7^ seconds (∼ 2 years), and the ratio of tumour cells and healthy cells, $\frac{\sum_{\phi >0}^{\Phi }\rho (\phi ,t^{⋆})}{\rho (0,t^{⋆})}$, is 0.002. For breast cancer cells with an averaged diameter of 50 *μ*m, the total amount of cancer cells at detection time is of the order 10^6^ [60]. Using the above estimated values, the maximum size of the simulated tumour is 5 cm.

**Fig 6:**
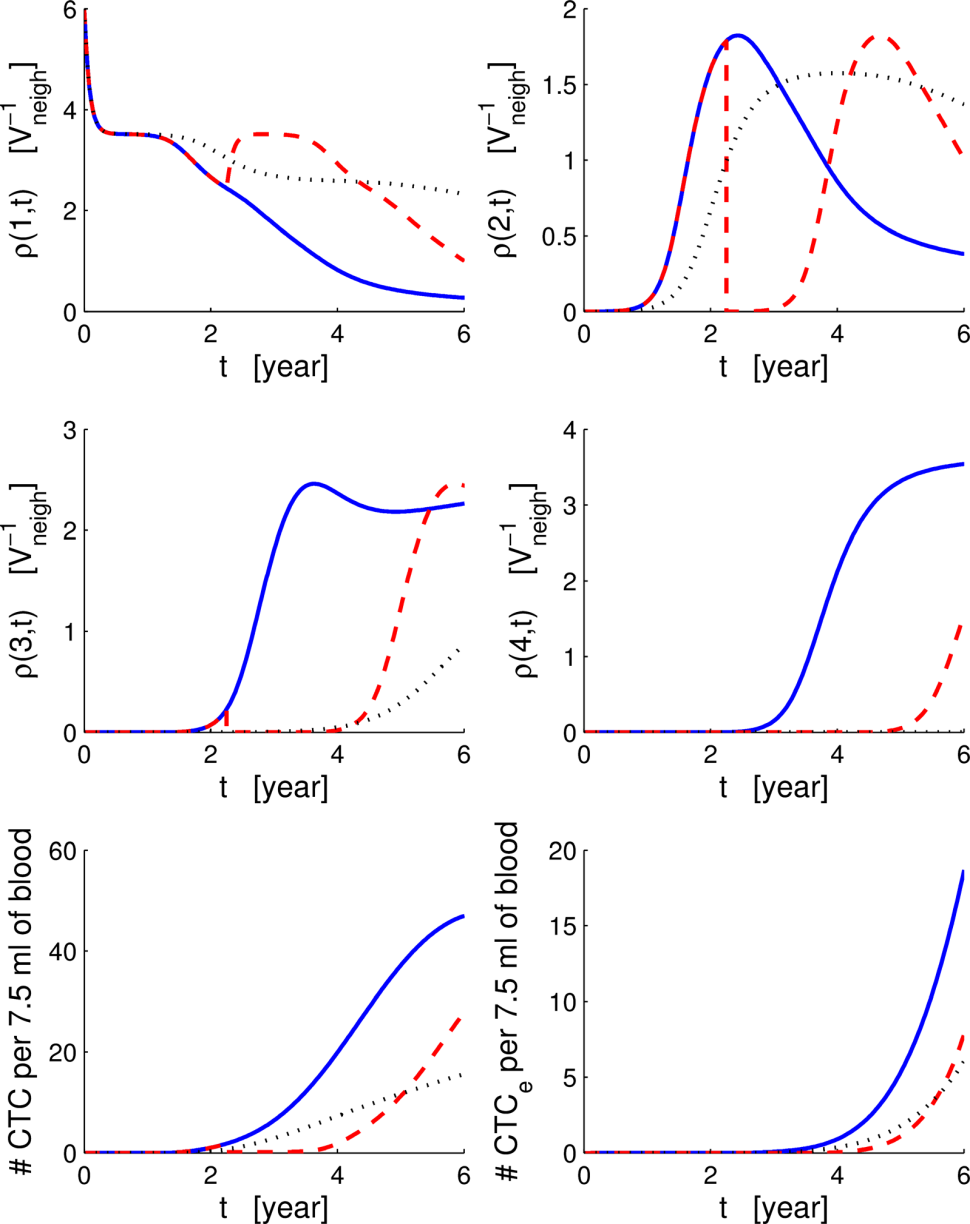
Breast, circulatory and bone compartments. Solution of the system of ODEs with and without treatment. From the top-left to the bottom-right, we show the solution of Eqs (3, 7) with *ϕ* from 1 to 4, Eqs (4, 8) and Eqs (5, 9). Continuous blue lines show the case without treatments, and dashed red lines shows the case with bisphosphonates administration. Dotted black lines show the case without TGF-*β* differences between cell phenotypes: $R_{\text{ec}}^{⋆}{\left(\phi \right)}^{g(\phi )}=R_{\text{ec}}^{⋆}{\left(1\right)}^{g(1)}$ for all *ϕ*. The time is in years.

The solutions of the system of Eqs (3–5) are shown in Fig 6. The parameters are all set to the averaged values as listed in Table 2. We observe that before the time of detection the concentration of cancer cells (*ρ*(1, *t*), *ρ*(2, *t*) and *ρ*(3, *t*)) in the breast is very small, and also the growth of the tumour is slow. Around the detection time, there is a change in the concentration of cancer cells which begins to increase rapidly. Aggressive cancer cells with *ϕ* = 3 have a concentration characterised by an initial plateau, corresponding to the time necessary to develop a TGF-*β* resistant phenotype, and a final plateau where the number of cancer cells are limited by a maximum capacity both in volume and resources. The CTCs generated by cancer cells in the breast begin to increase after tumoural and aggressive cancer cells develop; nevertheless, the growth of CTCs slows down due to the extravasation process and the response of the immune system against the CTCs. The decrease of pre-neoplastic cancer cells is due to the competition with more aggressive cancer cells *ρ*(2, *t*) and *ρ*(3, *t*).

As already discussed, TGF-*β* has a key role in the tumour evolution when aggressive cancer cells are present. To better understand this aspect, in Fig 6, we show the differences in the dynamics of the cancer when we neglected the effects of the TGF-*β*. We simulated the system of Eqs (3–5) under the condition that the TGF-*β* produced by all the cell sub-populations is set to the minimum value $R_{\text{ec}}^{⋆}(\phi )=R_{\text{ec}}^{⋆}(1)$, and we imposed all cells to respond to the TGF-*β* signaling in the same way $R_{\text{ec}}^{⋆}{\left(\phi \right)}^{g(\phi )}=R_{\text{ec}}^{⋆}{\left(1\right)}^{g(1)}$. Constraining all cells to produce the same minimal amount of TGF-*β* causes all four cell phenotypes to reach similar asymptotic steady values (∼ 1.7 for *ρ*(1) and *ρ*(2) and ∼ 2.3 for *ρ*(3) and *ρ*(4) based on their respective capacities, see Table 2) at times bigger than the simulated time *T*_*f*_ = 8*10^8^ *s*.

**Fig 7:**
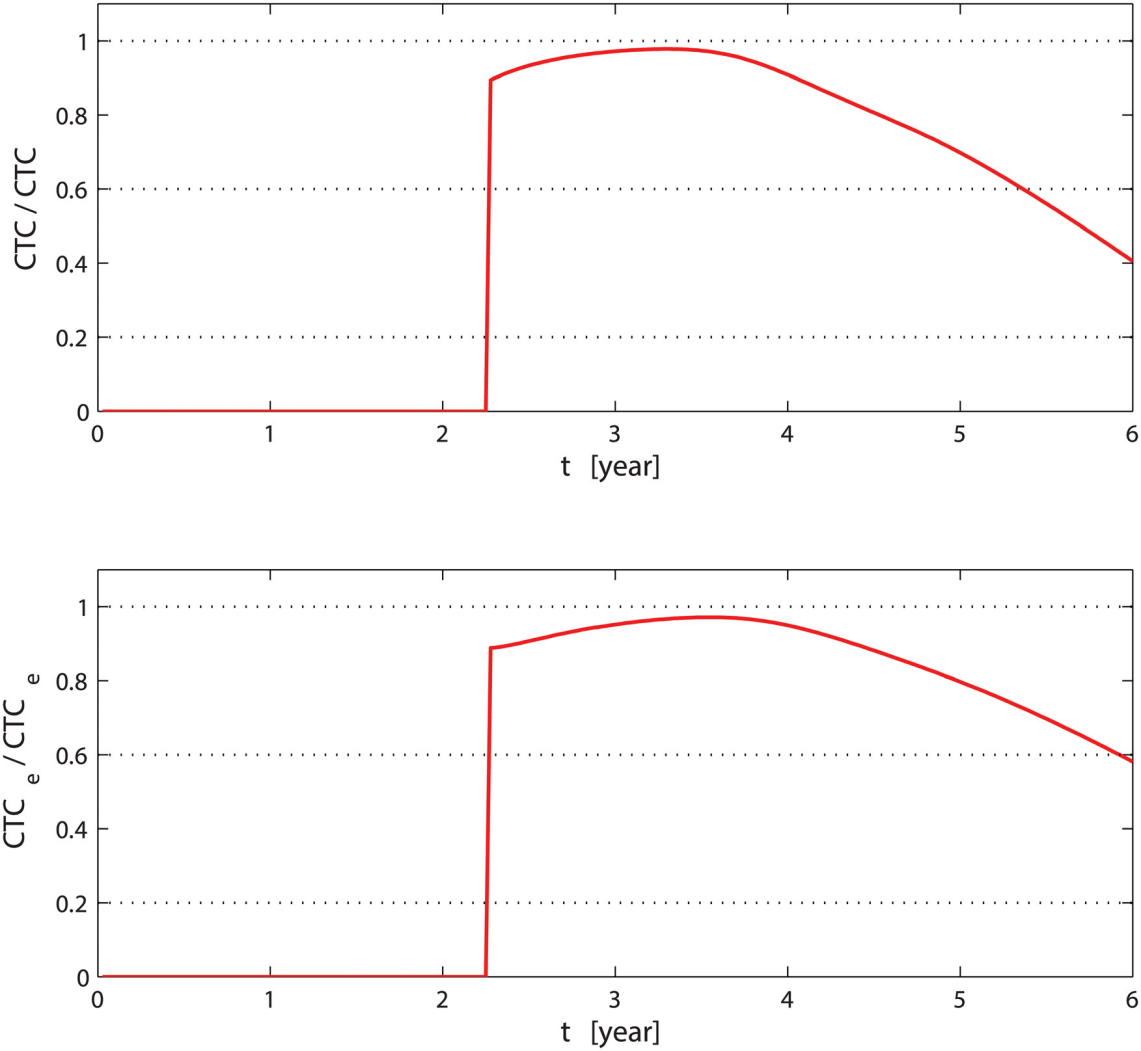
Relative variation of CTCs and extravasating CTCs. The top (bottom) graph shows the ratio between Δ*CTC* (Δ*CTC*_*e*_), which is defined as the amount of CTCs (*CTC*_*e*_) without therapy minus the CTCs (*CTC*_*e*_) when treatment is administered, and the amount of CTCs (*CTC*_*e*_) without therapy. The time on the abscissa is in years.

To have a complete picture of the multi-compartment model, we compare the system of ODEs Eqs (7–9) (bisphosphonates treatment) to the general case (no treatment) in order to understand how the drug affects the response. In the previous subsection, we have already seen how the administration of bisphosphonates affects the survival probability. In terms of mean field cell populations, we observe that healthy cells in the DLU (*ρ*(0, *t*)) are minimally affected by the bisphosphonates as indicated in [42]; on the contrary, healthy cells increase as a consequence of the reduction of competition between them and cancer cells. Pre-neoplastic and cancer cells in the mammary duct (*ρ*(1, *t*) and *ρ*(2, *t*)) drop immediately after the administration at time *t*_1_ forming a step. As shown in Fig 6, the development of new cancer cells, after the administration of bisphosphonate, is delayed, and the effect is more enhanced in the aggressive sub-population *ρ*(Φ, *t*_1_). The intensity of the step on the sub-populations *ρ*(*ϕ* > 0, *t*_1_) depends both on the quantity of drug administered per unit of time and on time *t*_1_. The bisphosphonates also delays the formation of CTCs. After the transition period following the treatment, the slope of the CTCs increases until it becomes parallel to the slope of the curve without drugs, while on the contrary, *CTC*_*e*_ recovering is slower than in the case without bisphosphonates. In Fig 7, we show the relative variations of CTCs and extravasating CTCs (*CTC*_*e*_) when we add the bisphosphonates compared with the case without treatment. The drug reduces the CTCs by more than 40%, and the extravasating CTCs are reduced by more than 60%; immediately after *t*_1_, both types of CTCs have a rapid drop of 90%. Considering that *CTC*_*e*_ are the only cells that metastasise, we can see that bisphosphonates have an effective role in contrasting the development of metastasis and delaying the death of the patients.

In the previous cases (with and without treatments), we have considered that metastases in the bone are originated only by breast cancer cells with four driver mutations relative to four proteins EPCAM, CD44, CD47 and MET, and they correspond to four branching of the CTCs departing from the DLU. An important question to address is why is it necessary to have only four driver mutations to develop metastasis? Is this a constraint related to the evolutionary process of cancer? There is no easy answer for these questions, but with our model, we can answer the following questions. What happens if, for some reason, there is a further driver mutation in the CTCs metastasisation process which has been neglected or has not yet been included? What happen if, instead, we overestimate the number of important driver mutations necessary to generate CTCs capable of forming metastasis in the bone niche? In order to answer these questions, let us consider the first special case where we modify Eqs (4–5), and we include a further factor $\frac{x_{j}}{x_{0}}$ in the extravasating flux corresponding to a generic driver mutation necessary to develop pro-metastatic behaviour as follows:

∂tCTC(t)=∑aaarintVROIΓ(CEPC)∑ϕ=1Φρ(ϕ,t)︷intravasatingCTCs−∑aaarimmCTC(t)Γ(C47¯−C47)︷CTCsdonotescapetheimmunesystem+−∑aaarextCTC(t)Γ(C44)Γ(C47)Γ(CMET)Γ(CGEN)︷extravasatingCTCs, (10)

∂tCTCe(t)=∑aaarextCTC(t)Γ(C44)Γ(C47)Γ(CMET)Γ(CGEN)︷extravasatingCTCs−∑aaarseedCTCe(t)︷seedingCTCs. (11)

where *C*_*GEN*_ is the average gene expression of a necessary protein. In our simulation, we set *C*_*GEN*_ equals to *C*_*MET*_ to give a possible example.

**Fig 8:**
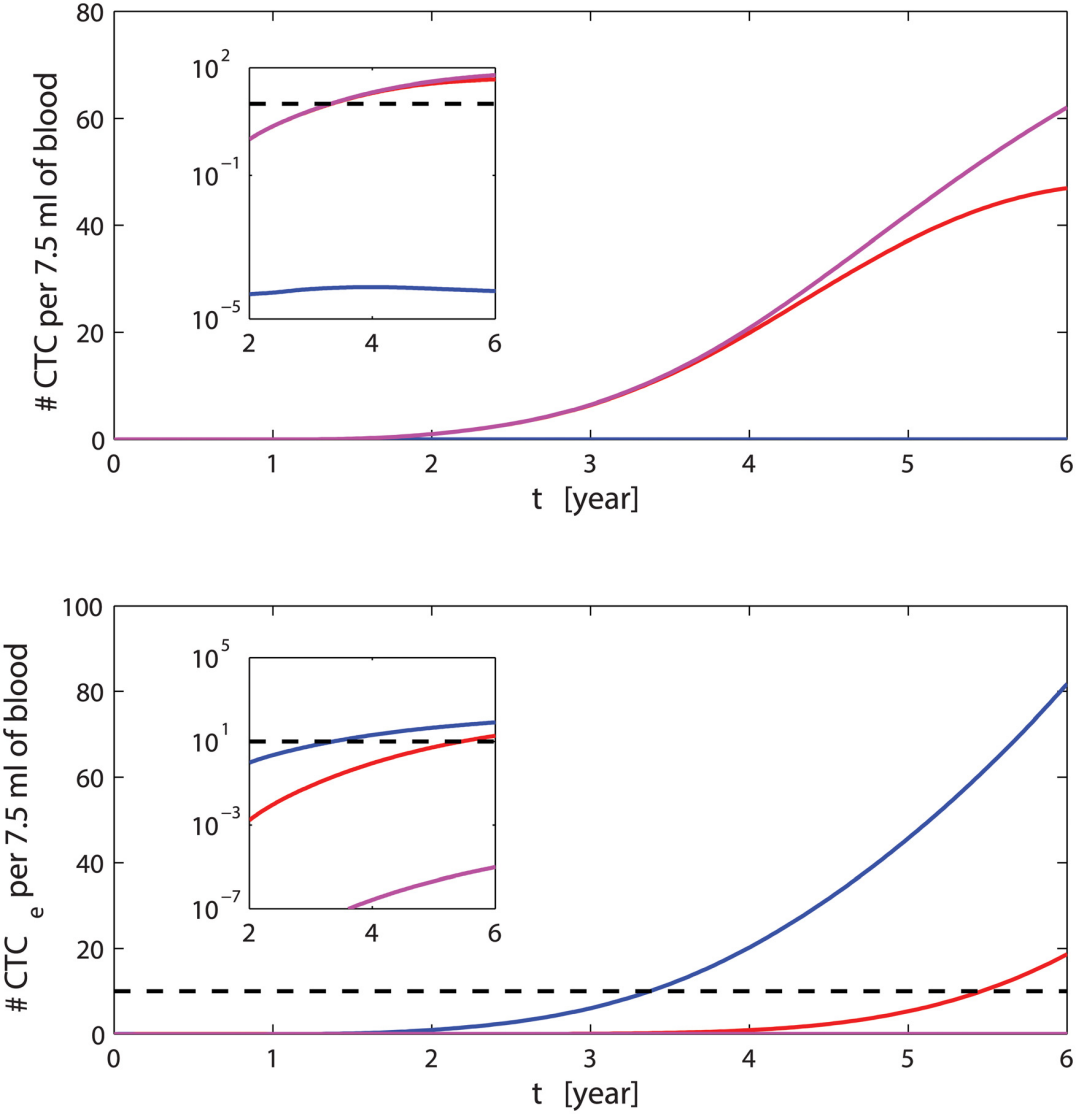
CTCs amount for different number of driver mutations. The red curves represents the dynamics of CTCs on top and *CTC*_*e*_ below when only two driver mutations (*CD*44 and *CD*47) are considered. The magenta lines describe the case of four driver mutations (*CD*44, *CD*47), *MET* and *GEN* = *MET*). The blue lines are the standard case with three mutations (*CD*44, *CD*47 and *MET*). The dashed line indicates 10 *CTC*_*e*_. The inset shows the differences in the amounts of *CTC*_*e*_ in semilog scale for the three cases.

**Fig 9:**
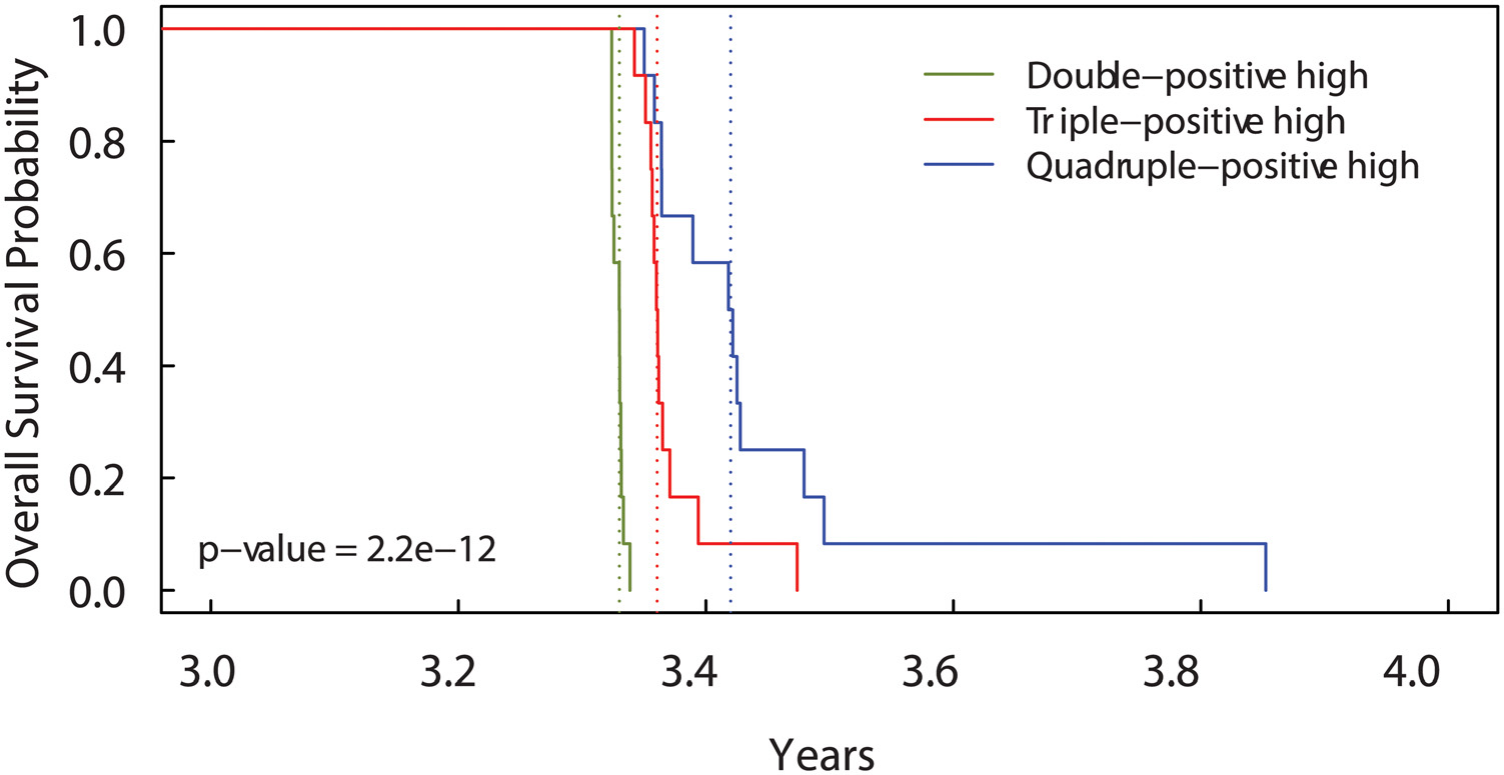
Kaplan-Meier curves for Overall Survival Probability on different numbers of genes. Patients with more than the median number of *CD*44^+^ *CD*47^+^ cells (“Double-positive high” patients) have shorter overall survival than patients with more than the median number of *CD*44^+^ *CD*47^+^ *MET* ^+^ cells (“Triple-positive hogh” patients). Patients with more than the median number of *CD*44^+^ *CD*47^+^ *MET* ^+^ *GEN* ^+^ cells (“Quadruple-positive high” patients with *GEN* = *MET*) have the longest overall survival. The dotted lines show the median overall survival times.

Solving the ODEs and comparing the results of the general case versus the special case with the modified ODEs Eqs (10–11)) in Fig 8, we see that after the time *t* ^⋆^, the number of CTCs remaining in the circulatory system is higher in the case with five driver mutations than in the case with four driver mutations. However, the further mutation causes a decrease of the number of *CTC*_*e*_ which remains lower than 10 units per 7.5 ml of blood. This means the development of metastasis with a further branching related to the protein GEN is much less probable, and in average, the time needed to obtain a sufficient number of CTCs for the formation of metastasis before the occurrence of death increases drastically. Also the overall survival probability (Fig 9), reflects the previous stated behaviours showing that the slope of this special case (“Quadruple-positive high”) is larger than in the general case.

Similarly, if we include only three driver mutations and, for example, we consider the effects of the MET protein unnecessary, than setting Γ(*C*_*MET*_) = 1 into Eqs (4) and (5), we see that the extravasating CTCs increase much faster than in the previous cases reaching 10 units per 7.5 ml of blood few months after the time of detection, see Fig 8; hence, the formation metastasis occurs too prematurely resulting in inconsistency with clinical survival times, see Fig 9.

Considering the experimental difficulties and costs, the mathematical multi-compartment model proposed here represents a predictive tool that helps to identify the minimum number of driver mutations involved in the formation of metastasis by linking it with the survival probability.

The attack of the immune system on the CTCs might change from person to person. The diversity, or the delay of the immune response given the same quantity of CTCs might result in metastasis formation even by cells with moderate or low CD47 protein on their surface. Hence, in the second special case, we address the delay of the immune system response by setting to zero the number of cells killed by the immune cells for the period of time [*t*_*i*1_, *t*_*i*2_]; therefore, defining the function Δ_*i*_(*t*) = [1−*H*(*t*−*t*_*i*1_)*H*(*t*_*i*2_−*t*)], Eqs (4) and (5) become:

∂tCTC(t)=∑aaarintVROIΓ(CEPC)∑ϕ=1Φρ(ϕ,t)︷intravasatingCTCs−∑aaarimmCTC(t)Γ[(C47¯−C47)Δi(t)]︷CTCsdonotescapetheimmunesystem+−∑aaarextCTC(t)Γ(C44)Γ(C47)︷extravasatingCTCs, (12)

∂tCTCe(t)=∑aaarextCTC(t)Γ(C44)Γ(C47)︷extravasatingCTCs−∑aaarseedCTCe(t)︷seedingCTCs. (13)

**Fig 10:**
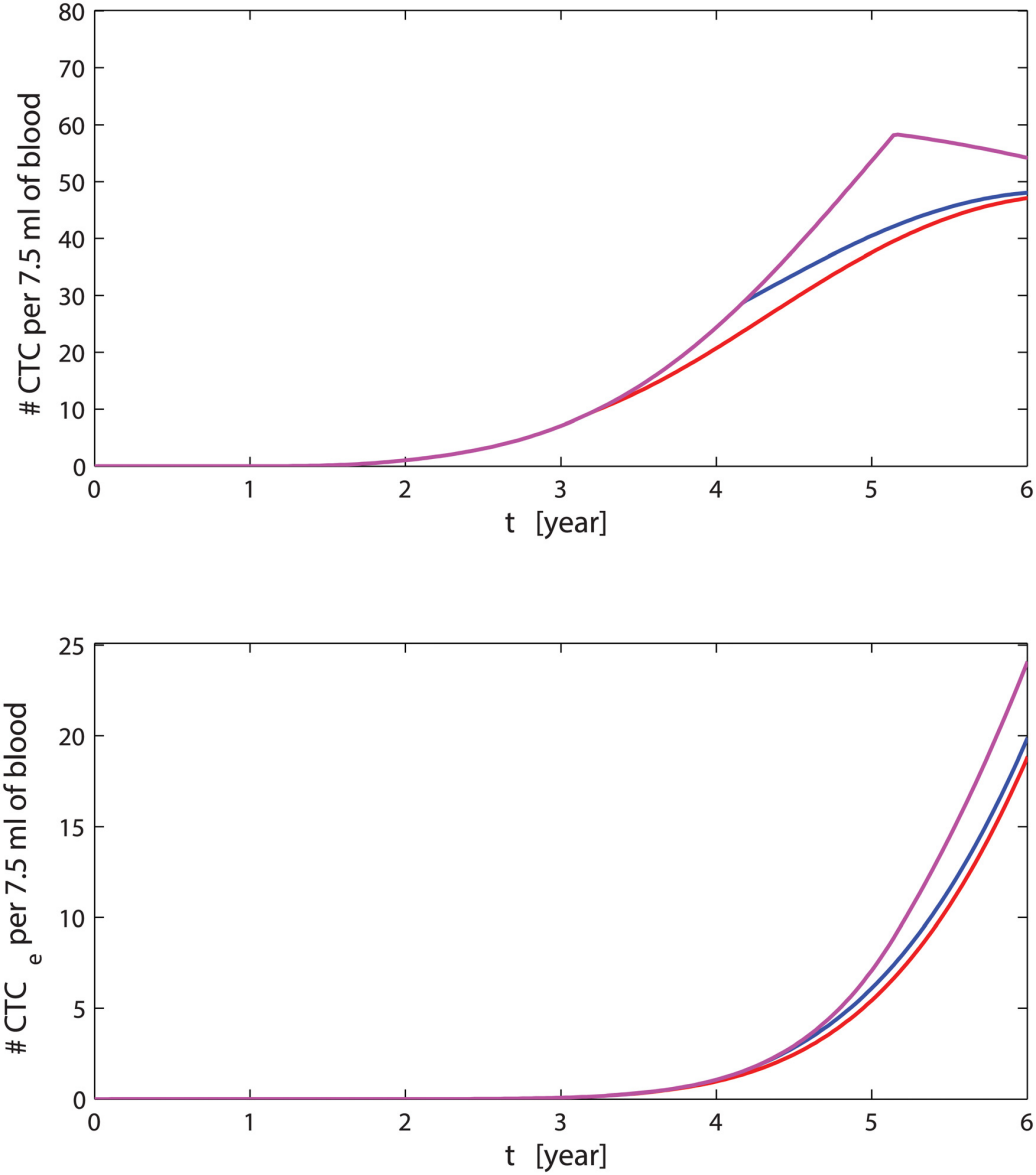
CTCs in presence of delayed immune response. Amount of CTCs and extravasating CTCs when the immune response peaks after 38 months (red lines), 50 months (blue lines) and 62 months (magenta lines). The time on the abscissa is in years.

In this case, we aim to propose a variation of the model to describe those sub-groups of patients which develop metastases at very early stages. In Fig 10, we see that the CTCs decrease occurs later in respect to the general case resulting in a higher amount of CTCs. The effects due to the reduced efficacy of the immune response in the interval of time [*t*_*i*1_, *t*_*i*2_] is even more evident in the increase of the extravasating CTCs. Delay in the immune response might depend on many factors, for example, a reduced sensitivity of the immune system to detect CTCs and distinguish the “self” from the “non-self”. Presence of other diseases or depression are factors that can overwhelm the response of the immune system and its promptness. The sensitivity of the immune system is independent from the amount of CD47, but depends on its threshold of detecting variations. This is why we included the model of this phenomenon separately as a special case.

## Discussion

We presented a mathematical model able to predict survival outcomes in metastatic breast cancer patients by using the gene expression profile of circulating cancer cells.

The proposed model emphasises the strong relationship between CTCs and survival probability in metastatic breast cancer. In particular, the model integrates different aspects of physiology (compartments), epidemiology (survival) and molecular information (gene expression data); this integration represents a semiquantitative but meaningful approach inching towards disease outcome predictability. The mathematical model contains novelties in all its parts such as the application of the branching models for molecular biomarkers in a dynamic model. TGF-*β* pathway and therapy couple several compartments; even drugs with a limited action on each compartment could have a larger effect on the whole system. This suggests that the administration of a cycling or multiple therapy could have even larger impact. Hence, this model could help understanding drug effectiveness in the breast-blood-bone system. Other aspects such as drug effectiveness for different therapy cycling administration or simultaneous administration of concurrent therapies could be incorporated. An important development of the model is the possibility to use molecular data and survival analysis to estimate how many unknown markers or how much the known markers should be overexpressed to give productive metastasis. In other words, if the number of productive markers are experimentally known, we can estimate the overexpression levels requires to match the CTCs heterogeneity. If they are not known we could estimate their number or their nature. Therefore, given molecular and survival data the model could be integrate with inferential approaches based on Cox network regression. We believe that the introduction of mathematical modelling of tumour microenvironments will help in bridging molecular and clinical evidences for bone metastasis derived from breast cancer. The model also provides a useful tool to predict survival probability under different conditions (CTCs expression levels and different immune responses). Recent studies have highlight controversial results of the use of mammography for cancer survival (known as “The Great Mammography Debate”, see [61–65]). As shown in this work, measuring CTCs in the blood stream can be an effective complementation to mammography. Therefore, we felt that the huge interest in CTC-based monitoring and therapies would benefit from this predictive mathematical model to analyse the probability of formation of CTCs and their interaction with different microenvironments. Moreover, the model and the software are effective in supporting hypothesis generation, mode of action understanding for candidate drugs, as well as supporting the construction of disease pathway interactions for different types of cancer and mathematical modelling for drug development projects.

## Methods

### Gene expression analysis

In order to set the gene expression values for the parameters *C*_*EPC*_, *C*_44_, *C*_47_ and *C*_*MET*_, we used four breast cancer microarray datasets downloaded from the Gene Expression Omnibus as raw. CEL files (accession numbers: GSE4525, GSE3494, GSE2034 and GSE6532). All the four datasets were generated by the Affymetrix HG-U133A platform. We averaged expression values for probes which map to the same gene and normalised each dataset with respect to one sample. Finally, we set *C*_*EPC*_, *C*_44_, *C*_47_ and *C*_*MET*_ equal to the average gene expression value (1.0828, 1.0363, 1.0971 and 1.0828 respectively). Moreover, we set $\bar{C_{47}}$ (the CD47 alert threshold) as the maximum value of *CD*47 gene expression level among the four datasets ($\bar{C_{47}}$ = 3.2976). This threshold is useful to regulate the effect of the immune system in Eq (4), Eq (8), Eq (10) and Eq (12). Indeed, we need an upper-bound for the *C*_47_ expression level so that the difference $\bar{C_{47}}-C_{47}$ provides a measure of the CD47 expression level. Higher is the difference $\bar{C_{47}}-C_{47}$, lower is the probability of evading the immune control. Further statistical analysis to characterise the overexpression of these markers (statistical significance tests) are reported in the Supporting Information (S4 Text and S1 Table) and in [66–75].

For the estimation of the parameters relative to the synthesis of TGF-*β*, the expression of their receptors and the amount of internalised TGF-*β*, we have used the model in [2] and analysed the following datasets (accession numbers): GSE14548, GSE33450 and GSE8977. These datasets originate from experimental design on early stages breast cancer progression and tumour microenvironment. The raw files were processed and normalised individually by RMA package and library files provided by the Bioconductor project [76]. The Bioconductor package limma was also used to calculate average expression levels. We have used gene expression averaged quantities to better unveil the functions of the TGF-*β* in the cancer dynamics.

## Supporting Information

S1 Text Branching process. (PDF)

S2 Text Full set of model equations. (PDF)

S3 Text Sensitivity analysis. (PDF)

S4 Text Data analysis. (PDF)

S1 Table List of datasets. Details of the datasets analysed to estimate the p-value of overexpression of CD47, CD44 and EPCAM in ductal carcinoma in situ (DCIS) and invasive ductal carcinoma (IDC) versus control under different experimental conditions.
(PDF)

S1 Fig Sensitivity analysis for *ρ*(0, *t*) before bisphosphonate administration. On the left side, the histogram of the PRCC values with p-values < 10^−4^ between *ρ*(0, *t*) and the parameters computed at time *t* = 600 days after the development of the disease. The yellow bar indicates the non-significant PRCCs range. The listed parameters are all those whose PRCC values cross the non-significant PRCCs range at least one time during the simulation time interval. On the right side, the scatter plots of the parameters with the highest PRCCs.
(EPS)

S2 Fig Sensitivity analysis for *ρ*(1, *t*) before bisphosphonate administration. On the left side, the histogram of the PRCC values with p-values < 10^−4^ between *ρ*(1, *t*) and the parameters computed at time *t* = 600 days after the development of the disease. The yellow bar indicates the non-significant PRCCs range. The listed parameters are all those whose PRCC values cross the non-significant PRCCs range at least one time during the simulation time interval. On the right side, the scatter plots of the parameters with the highest PRCCs.
(EPS)

S3 Fig Sensitivity analysis for *ρ*(2, *t*) before bisphosphonate administration. On the left side, the histogram of the PRCC values with p-values < 10^−4^ between *ρ*(2, *t*) and the parameters computed at time *t* = 600 days after the development of the disease. The yellow bar indicates the non-significant PRCCs range. The listed parameters are all those whose PRCC values cross the non-significant PRCCs range at least one time during the simulation time interval. On the right side, the scatter plots of the parameters with the highest PRCCs.
(EPS)

S4 Fig Sensitivity analysis for *ρ*(3, *t*) before bisphosphonate administration. On the left side, the histogram of the PRCC values with p-values < 10^−4^ between *ρ*(3, *t*) and the parameters computed at time *t* = 600 days after the development of the disease. The yellow bar indicates the non-significant PRCCs range. The listed parameters are all those whose PRCC values cross the non-significant PRCCs range at least one time during the simulation time interval. On the right side, the scatter plots of the parameters with the highest PRCCs.
(EPS)

S5 Fig Sensitivity analysis for *CTC*(*t*) before bisphosphonate administration. On the left side, the histogram of the PRCC values with p-values < 10^−4^ between *CTC*(*t*) and the parameters computed at time *t* = 600 days after the development of the disease. The yellow bar indicates the non-significant PRCCs range. The listed parameters are all those whose PRCC values cross the non-significant PRCCs range at least one time during the simulation time interval. On the right side, the scatter plots of the parameters with the highest PRCCs.
(EPS)

S6 Fig Sensitivity analysis for *CTC_e_*(*t*) before bisphosphonate administration. On the left side, the histogram of the PRCC values with p-values < 10^−4^ between *CTC*_*e*_(*t*) and the parameters computed at time *t* = 600 days after the development of the disease. The yellow bar indicates the non-significant PRCCs range. The listed parameters are all those whose PRCC values cross the non-significant PRCCs range at least one time during the simulation time interval. On the right side, the scatter plots of the parameters with the highest PRCCs.
(EPS)

S7 Fig Sensitivity analysis for *ρ*(0, *t*) after bisphosphonate administration. On the left side, the histogram of the PRCC values with p-values < 10^−4^ between *ρ*(0, *t*) and the parameters computed at time *t* = 1130 days after the development of the disease. The yellow bar indicates the non-significant PRCCs range. The listed parameters are all those whose PRCC values cross the non-significant PRCCs range at least one time during the simulation time interval. On the right side, the scatter plots of the parameters with the highest PRCCs.
(EPS)

S8 Fig Sensitivity analysis for *ρ*(1, *t*) after bisphosphonate administration. On the left side, the histogram of the PRCC values with p-values < 10^−4^ between *ρ*(1, *t*) and the parameters computed at time *t* = 1130 days after the development of the disease. The yellow bar indicates the non-significant PRCCs range. The listed parameters are all those whose PRCC values cross the non-significant PRCCs range at least one time during the simulation time interval. On the right side, the scatter plots of the parameters with the highest PRCCs.
(EPS)

S9 Fig Sensitivity analysis for *ρ*(2, *t*) after bisphosphonate administration. On the left side, the histogram of the PRCC values with p-values < 10^−4^ between *ρ*(2, *t*) and the parameters computed at time *t* = 1130 days after the development of the disease. The yellow bar indicates the non-significant PRCCs range. The listed parameters are all those whose PRCC values cross the non-significant PRCCs range at least one time during the simulation time interval. On the right side, the scatter plots of the parameters with the highest PRCCs.
(EPS)

S10 Fig Sensitivity analysis for *ρ*(3, *t*) after bisphosphonate administration. On the left side, the histogram of the PRCC values with p-values < 10^−4^ between *ρ*(3, *t*) and the parameters computed at time *t* = 1130 days after the development of the disease. The yellow bar indicates the non-significant PRCCs range. The listed parameters are all those whose PRCC values cross the non-significant PRCCs range at least one time during the simulation time interval. On the right side, the scatter plots of the parameters with the highest PRCCs.
(EPS)

S11 Fig Sensitivity analysis for *CTC*(*t*) after bisphosphonate administration. On the left side, the histogram of the PRCC values with p-values < 10^−4^ between *CTC*(*t*) and the parameters computed at time *t* = 1130 days after the development of the disease. The yellow bar indicates the non-significant PRCCs range. The listed parameters are all those whose PRCC values cross the non-significant PRCCs range at least one time during the simulation time interval. On the right side, the scatter plots of the parameters with the highest PRCCs.
(EPS)

S12 Fig Sensitivity analysis for *CTC_e_*(*t*) after bisphosphonate administration. On the left side, the histogram of the PRCC values with p-values < 10^−4^ between *CTC*_*e*_(*t*) and the parameters computed at time *t* = 1130 days after the development of the disease. The yellow bar indicates the non-significant PRCCs range. The listed parameters are all those whose PRCC values cross the non-significant PRCCs range at least one time during the simulation time interval. On the right side, the scatter plots of the parameters with the highest PRCCs.
(EPS)

S1 Code Branching Process Model code. (ZIP)
