# Supplementary material for: Modelling Circulating Tumour Cells for Personalised Survival Prediction in Metastatic Breast Cancer
Source: PLoS Comput Biol. 2015 May 15;11(5):e1004199. doi: 10.1371/journal.pcbi.1004199 (PMC4433130; doi:10.1371/journal.pcbi.1004199)
Supplement: S1 Text — (PDF) [file pcbi.1004199.s001.pdf]

Modelling circulating tumour cells for personalised survival prediction in metastatic breast cancer

Gianluca Ascolani[1,◕], Annalisa Occhipinti[1,◕*], Pietro Liò[1,◕]

1 University of Cambridge, Computer Laboratory, Cambridge, UK

◕These authors contributed equally to this work. * ao356@cl.cam.ac.uk

S1 Text: Branching Process

In our model, we are interested in the number of cells expressing the mutations leading to metastatic behaviour (CD44, CD47 and MET mutations [1]). In particular, we focus on the following questions: how many cells in the tumour develop one given mutation? How many cells can present simultaneously all the three mutations? Such multi-mutation path can be obtained through several *paths*, where a path is a sequence of mutations leading from $\mathbf{j} = (0,0,0)$ (no mutation) to $\mathbf{j}' = (1,1,1)$ (all the three genes are mutated). For the sake of simplicity, let us assume that mutation at position 1 is related to CD44, at position 2 is related to MET and position 3 describes the mutation of CD47. An example of path leading to full mutation is $\mathcal{P} = (0,0,0) \rightarrow (1,0,1) \rightarrow (1,1,1)$ (mutations of CD47 and CD44 happen simultaneously first, then MET mutation takes place). As a consequence, this mutation process can be described by the branching process presented in [2]. We assume that only one mutation can happen at once and if a mutation has been acquired it is not possible to go back. So, path like (0,0,0) → (1,0,1) → (0,0,1) are not accepted.

Following the terminology used in [2], the profiles along a path are indexed as k, the number of potential mutations that would lead from profile $k-1$ to k, along a particular path, is ν_k, while the number of mutations leading from profile $k-1$ to a profile not on the path is denoted as η_k. For example, in the path $(0,0,0) \rightarrow (1,0,1) \rightarrow (1,1,1)$ we have

$$\begin{aligned} \nu_1 &= n_1 + n_3 \\ \eta_1 &= n_2 \\ \nu_2 &= n_2 \\ \eta_2 &= 0 \end{aligned}$$

We are interested in the number of cells with a given mutation. Let us define $x_{\mathbf{j}}(t)$ as the expected number of cells of profile $\mathbf{j}$ at time t, for each profile $\mathbf{j} = (j_1, ..., j_m)$ in a m-profile path. These expected numbers satisfy the following system of differential equations [2]:

$$x'_j(t) = [r - r_b(\eta_{j+1} + \nu_{j+1})\ u_0]x_j + r_b\ \eta_j\ u_0\ x_{j-1} \tag{1}$$

where r_b is the cell birth rate, r_d is the cell death rate, u_0 is the point mutation rate and $r = r_b(1-u_0) - r_d$. According to [2], we set $x_{-1} = 0$ for all t, $\eta_0 = \nu_0 = \eta_{m+1} = \nu_{m+1} = 0$ and initial conditions as $x_0(0) = 1$ and $x_j(0) = 0$ for $j = 1, ..., m$.
From profile $\mathbf{j} = (0,0,0)$ to $\mathbf{j}' = (1,1,1)$ there are six different paths.

Path 1 (0,0,0) → (1,0,0) → (1,1,0) → (1,1,1),
with $\nu_1 = n_1,\ \eta_1 = n_2 + n_3,\ \nu_2 = n_2,\ \eta_2 = n_3,\ \nu_3 = n_3,\ \eta_3 = 0;$

Path 2 (0,0,0) $\rightarrow$ (1,0,0) $\rightarrow$ (1,0,1) $\rightarrow$ (1,1,1),
with $\nu_1 = n_1,\ \eta_1 = n_2 + n_3,\ \nu_2 = n_3,\ \eta_2 = n_2,\ \nu_3 = n_2,\ \eta_3 = 0$;

Path 3 (0,0,0) $\rightarrow$ (0,1,0) $\rightarrow$ (1,1,0) $\rightarrow$ (1,1,1),
with $\nu_1 = n_2,\ \eta_1 = n_1 + n_3,\ \nu_2 = n_1,\ \eta_2 = n_3,\ \nu_3 = n_3,\ \eta_3 = 0$;

Path 4 (0,0,0) $\rightarrow$ (0,1,0) $\rightarrow$ (0,1,1) $\rightarrow$ (1,1,1),
with $\nu_1 = n_2,\ \eta_1 = n_1 + n_3,\ \nu_2 = n_3,\ \eta_2 = n_1,\ \nu_3 = n_1,\ \eta_3 = 0$;

Path 5 (0,0,0) $\rightarrow$ (0,0,1) $\rightarrow$ (1,0,1) $\rightarrow$ (1,1,1),
with $\nu_1 = n_3,\ \eta_1 = n_1 + n_2,\ \nu_2 = n_1,\ \eta_2 = n_2,\ \nu_3 = n_1,\ \eta_3 = 0$;

Path 6 (0,0,0) $\rightarrow$ (0,0,1) $\rightarrow$ (0,1,1) $\rightarrow$ (1,1,1),
with $\nu_1 = n_3,\ \eta_1 = n_1 + n_2,\ \nu_2 = n_2,\ \eta_2 = n_1,\ \nu_3 = n_1,\ \eta_3 = 0$;

Each path can be described by the following system:

$$X'(t) = A_k X(t), \quad k = 1, ..., 6 \tag{2}$$

where

$$X'(t) = \begin{pmatrix} x'_{(0,0,0)}(t) \\ x'_{(1,0,0)}(t) \\ x'_{(0,1,0)}(t) \\ x'_{(0,0,1)}(t) \\ x'_{(1,1,0)}(t) \\ x'_{(1,0,1)}(t) \\ x'_{(0,1,1)}(t) \\ x'_{(1,1,1)}(t) \end{pmatrix}, \qquad X(t) = \begin{pmatrix} x_{(0,0,0)}(t) \\ x_{(1,0,0)}(t) \\ x_{(0,1,0)}(t) \\ x_{(0,0,1)}(t) \\ x_{(1,1,0)}(t) \\ x_{(1,0,1)}(t) \\ x_{(0,1,1)}(t) \\ x_{(1,1,1)}(t) \end{pmatrix}$$

and A_k for $k = 1, ..., 6$ is the matrix describing the numbers of "on–path" and "off–path" mutations of the k-th path as follows (here we report only A_1 and A_2, but all the other cases can be easily obtained):

$$A_1 = \begin{pmatrix} r - r_b(u_1 + u_2 + u_3) & 0 & 0 & 0 & 0 & 0 & 0 & 0 \\ r_b u_1 & r - r_b(u_2 + u_3) & 0 & 0 & 0 & 0 & 0 & 0 \\ 0 & 0 & 0 & 0 & 0 & 0 & 0 & 0 \\ 0 & 0 & 0 & 0 & 0 & 0 & 0 & 0 \\ 0 & r_b u_2 & 0 & 0 & r - r_b u_3 & 0 & 0 & 0 \\ 0 & 0 & 0 & 0 & 0 & 0 & 0 & 0 \\ 0 & 0 & 0 & 0 & 0 & 0 & 0 & 0 \\ 0 & 0 & 0 & 0 & r_b u_3 & 0 & 0 & r \end{pmatrix}$$

$$A_2 = \begin{pmatrix} r - r_b(u_1 + u_2 + u_3) & 0 & 0 & 0 & 0 & 0 & 0 & 0 \\ r_b u_1 & r - r_b(u_2 + u_3) & 0 & 0 & 0 & 0 & 0 & 0 \\ 0 & 0 & 0 & 0 & 0 & 0 & 0 & 0 \\ 0 & 0 & 0 & 0 & 0 & 0 & 0 & 0 \\ 0 & 0 & 0 & 0 & 0 & 0 & 0 & 0 \\ 0 & r_b u_3 & 0 & 0 & 0 & r - r_b u_2 & 0 & 0 \\ 0 & 0 & 0 & 0 & 0 & 0 & 0 & 0 \\ 0 & 0 & 0 & 0 & 0 & r_b u_2 & 0 & r \end{pmatrix}$$

where $u_i = u\, n_i$ is the point mutation rate related to the ith gene.

The cell mutations from profile $\mathbf{j} = (0,0,0)$ to $\mathbf{j}' = (1,1,1)$ can follow the first path or the second one or the third one and so on. Hence, the system describing all the path possibilities is given by $X' = AX$, where $A = \sum_{k=1}^{6} A_k$. Moreover, each profile has to be taken into account only once, since each cell goes through only one given path with no repeated profiles. However, several profiles are encountered in more than one path (e.g., profile (0,0,0) is repeated in every paths and (1,0,0) is repeated in path 1 and 2). As a consequence, matrix A needs to be rescaled in order to avoid profile repetitions. Finally, the final system describing the whole branching process is the following

$$X' = S \circ AX \tag{3}$$

where $\circ$ is the Hadamard product or element–by–element product and S is the scaling matrix.

$$S = \begin{pmatrix} 1/6 & 1 & 1 & 1 & 1 & 1 & 1 & 1 \\ 1/2 & 1/2 & 1 & 1 & 1 & 1 & 1 & 1 \\ 1/2 & 1 & 1/2 & 1 & 1 & 1 & 1 & 1 \\ 1/2 & 1 & 1 & 1/2 & 1 & 1 & 1 & 1 \\ 1 & 1 & 1 & 1 & 1/2 & 1 & 1 & 1 \\ 1 & 1 & 1 & 1 & 1 & 1/2 & 1 & 1 \\ 1 & 1 & 1 & 1 & 1 & 1 & 1/2 & 1 \\ 1 & 1 & 1 & 1 & 1/2 & 1/2 & 1/2 & 1/6 \end{pmatrix}$$

$$S \circ A = \begin{pmatrix} r - r_b(u_1+u_2+u_3) & 0 & 0 & 0 & 0 & 0 & 0 & 0 \\ r_b u_1 & r - r_b(u_2+u_3) & 0 & 0 & 0 & 0 & 0 & 0 \\ r_b u_2 & 0 & r - r_b(u_1+u_3) & 0 & 0 & 0 & 0 & 0 \\ r_b u_3 & 0 & 0 & r - r_b(u_1+u_2) & 0 & 0 & 0 & 0 \\ 0 & r_b u_2 & r_b u_1 & 0 & r - r_b u_3 & 0 & 0 & 0 \\ 0 & r_b u_3 & 0 & r_b u_1 & 0 & r - r_b u_2 & 0 & 0 \\ 0 & 0 & r_b u_3 & r_b u_2 & 0 & 0 & r - r_b u_1 & 0 \\ 0 & 0 & 0 & 0 & r_b u_3 & r_b u_2 & r_b u_1 & r \end{pmatrix}$$

The explicit form of system of ODEs 3 is

$$\begin{cases} x'_{(0,0,0)}(t) = [r - r_b(u_1+u_2+u_3)]x_{(0,0,0)}(t) \\ x'_{(1,0,0)}(t) = r_b\ u_1 x_{(0,0,0)}(t) + [r - r_b(u_2+u_3)]x_{(1,0,0)}(t) \\ x'_{(0,1,0)}(t) = r_b u_2 x_{(0,0,0)}(t) + [r - r_b(u_1+u_3)]x_{(0,1,0)}(t) \\ x'_{(0,0,1)}(t) = r_b u_3 x_{(0,0,0)}(t) + [r - r_b(u_1+u_2)]x_{(0,1,0)}(t) \\ x'_{(1,1,0)}(t) = r_b u_2 x_{(1,0,0)}(t) + r_b u_1 x_{(0,1,0)}(t) + (r - r_b u_3)x_{(1,1,0)}(t) \\ x'_{(1,0,1)}(t) = r_b u_3 x_{(1,0,0)}(t) + r_b u_1 x_{(0,0,1)}(t) + (r - r_b u_2)x_{(1,0,1)}(t) \\ x'_{(0,1,1)}(t) = r_b u_3 x_{(0,1,0)}(t) + r_b u_2 x_{(0,0,1)}(t) + (r - r_b u_1)x_{(0,1,1)}(t) \\ x'_{(1,1,1)}(t) = r_b\ [u_3 x_{(1,1,0)}(t) + u_2 x_{(1,0,1)}(t) + u_1 x_{(0,1,1)}(t)] + r\ x_{(1,1,1)}(t) \end{cases}$$

The solution of system of ODEs 3 is given by

$$X(t) = \begin{pmatrix} x_{(0,0,0)}(t) = e^{[r-r_b(u_1+u_2+u_3)]t} \\ x_{(1,0,0)}(t) = e^{[r-r_b(u_1+u_2+u_3)]t}(e^{r_b u_1 t} - 1) \\ x_{(0,1,0)}(t) = e^{[r-r_b(u_1+u_2+u_3)]t}(e^{r_b u_2 t} - 1) \\ x_{(0,0,1)}(t) = e^{[r-r_b(u_1+u_2+u_3)]t}(e^{r_b u_3 t} - 1) \\ x_{(1,1,0)}(t) = e^{[r-r_b(u_1+u_2+u_3)]t}(e^{r_b u_1 t} - 1)(e^{r_b u_2 t} - 1) \\ x_{(1,0,1)}(t) = e^{[r-r_b(u_1+u_2+u_3)]t}(e^{r_b u_1 t} - 1)(e^{r_b u_3 t} - 1) \\ x_{(0,1,1)}(t) = e^{[r-r_b(u_1+u_2+u_3)]t}(e^{r_b u_2 t} - 1)(e^{r_b u_3 t} - 1) \\ x_{(1,1,1)}(t) = e^{[r-r-b(u_1+u_2+u_3)]t}(e^{r_b u_1 t} - 1)(e^{r_b u_2 t} - 1)(e^{r_b u_3 t} - 1) \end{pmatrix} \tag{4}$$

Our goal is knowing the number of cells with one given mutation at time t. If $\overline{x}_1(t)$ is the number of cells having CD44 mutation at time t, it follows that $\overline{x_1}(t) = x_{(1,0,0)}(t) + x_{(1,1,0)}(t) + x_{(1,0,1)}(t) + x_{(1,1,1)}(t)$. Analogously, $\overline{x_2}(t) = x_{(0,1,0)}(t) + x_{(1,1,0)}(t) + x_{(0,1,1)}(t) + x_{(1,1,1)}(t)$ is the number of cells with MET mutation and $\overline{x_3}(t) = x_{(0,0,1)}(t) + x_{(1,0,1)}(t) + x_{(0,1,1)}(t) + x_{(1,1,1)}(t)$ is the number of cells with CD47 mutation. Replacing solution 4 in the previous equations, we obtain

$$\begin{aligned}\overline{x_1}(t) &= e^{rt}(1 - e^{-r_b u_1 t})\\ \overline{x_2}(t) &= e^{rt}(1 - e^{-r_b u_2 t})\\ \overline{x_3}(t) &= e^{rt}(1 - e^{-r_b u_3 t})\end{aligned}$$

In conclusion, the general form of the branching process expressing the number of cells with mutation i, $i = CD44, MET, CD47$ at time t is

$$\overline{x_i}(t) = e^{rt}(1 - e^{-r_b u_i t})$$

where $u_i = u\, n_i$ is the point mutation rate related to the ith gene.

References

1. Baccelli I, Schneeweiss A, Riethdorf S, Stenzinger A, Schillert A, Vogel V, et al. Identification of a population of blood circulating tumor cells from breast cancer patients that initiates metastasis in a xenograft assay. Nat Biotech. 2013 Jun;31(6):539–544. Research. Available from: http://dx.doi.org/10.1038/nbt.2576.
2. Bozic I, Reiter JG, Allen B, Antal T, Chatterjee K, Shah P, et al. Evolutionary dynamics of cancer in response to targeted combination therapy. eLife. 2013;2.
