# Supplementary material for: Modelling Circulating Tumour Cells for Personalised Survival Prediction in Metastatic Breast Cancer
Source: PLoS Comput Biol. 2015 May 15;11(5):e1004199. doi: 10.1371/journal.pcbi.1004199 (PMC4433130; doi:10.1371/journal.pcbi.1004199)
Supplement: S2 Text — (PDF) [file pcbi.1004199.s002.pdf]

Modelling circulating tumour cells for personalised survival prediction in metastatic breast cancer

Gianluca Ascolani[1,◕], Annalisa Occhipinti[1,◕*], Pietro Liò[1,◕]

1 University of Cambridge, Computer Laboratory, Cambridge, UK

◕These authors contributed equally to this work. * ao356@cl.cam.ac.uk

S2 Text: Full set of model equations

General case: absence of medical treatments

- cell populations in the mammary duct

$$\partial_t \rho(\phi,t) = \overbrace{r_{\mathrm{p}} \left(1 - \delta_{\phi,0}\frac{\tilde{\rho}_0}{C_\phi} - \sum_{\substack{0\le\eta\le\Phi\\ \eta\neq\phi}} \frac{\rho(\eta,t)}{C_\eta}\right)\left(1 - \sum_{0\le\eta\le\Phi}\frac{\rho(\eta,t)}{C_\eta}\right)\frac{\rho(\phi,t)}{R^\star_{\mathrm{ec}}(\phi)^{g(\phi)}}}^{\text{symmetric cell proliferation}} - \overbrace{r_{\mathrm{a}} R^\star_{\mathrm{ec}}(\phi)^{g(\phi)}\rho(\phi,t)}^{\text{TGF-}\beta\text{ induced apoptosis}}$$
$$+ \overbrace{r_{\mathrm{m}}\left[(1-\delta_{\phi,0})\rho(\phi-1,t) - (1-\delta_{\phi,\Phi})\rho(\phi,t)\right]}^{\text{cell mutation}} - \overbrace{r_{int}\ (1-\delta_{\phi,0})\rho(\phi,t)\ \Gamma(C_{EPC})}^{\text{cells entering the blood stream}}$$

- CTC in the circulatory system

$$\partial_t CTC(t) = \overbrace{r_{int}\ V_{ROI}\sum_{\phi=1}^{\Phi}\rho(\phi,t)\ \Gamma(C_{EPC})}^{\text{intravasating CTCs}} - \overbrace{r_{imm}\ CTC(t)\ \Gamma(\overline{C_{47}} - C_{47})}^{\text{CTCs do not escape the immune system}} +$$
$$\overbrace{- r_{ext}\ CTC(t)\ \Gamma(C_{44})\ \Gamma(C_{47})\ \Gamma(C_{MET})}^{\text{extravasating CTCs}}$$

- CTC_e in the bone niche

$$\partial_t CTC_e(t) = \overbrace{r_{ext}\ CTC(t)\ \Gamma(C_{44})\ \Gamma(C_{47})\ \Gamma(C_{MET})}^{\text{extravasating CTCs}} - \overbrace{r_{seed}\ CTC_e(t)}^{\text{seeding CTCs}}$$

Special case: bisphosphonate treatment

- cell populations in the mammary duct

$$\partial_t \rho(\phi,t) = \overbrace{r_{\mathrm{p}} \left(1-\delta_{\phi,0}\frac{\tilde{\rho}_0}{C_\phi} - \sum_{\substack{0\le\eta\le\Phi\\ \eta\neq\phi}} \frac{\rho(\eta,t)}{C_\eta}\right)\left(1-\sum_{0\le\eta\le\Phi}\frac{\rho(\eta,t)}{C_\eta}\right)\frac{\rho(\phi,t)}{R^\star_{\mathrm{ec}}(\phi)^{g(\phi)}}}^{\text{symmetric cell proliferation}} - \overbrace{r_{\mathrm{a}} R^\star_{\mathrm{ec}}(\phi)^{g(\phi)}\rho(\phi,t)}^{\text{TGF-}\beta\text{ induced apoptosis}}$$

$$+ \overbrace{r_{\mathrm{m}}\left[(1-\delta_{\phi,0})\rho(\phi-1,t)-(1-\delta_{\phi,\Phi})\rho(\phi,t)\right]}^{\text{cell mutation}} - \overbrace{r_{int}\ (1-\delta_{\phi,0})\rho(\phi,t)\ \Gamma(C_{EPC})}^{\text{cells entering the blood stream}} - \overbrace{r_{BP}\ BP(t)\ \rho(\phi,t)}^{\text{therapy effect}}$$

- CTC in the circulatory system

$$\partial_t CTC(t) = \overbrace{r_{int}\ V_{ROI}\ \Gamma(C_{EPC})\ \sum_{\phi=1}^{\Phi}\rho(\phi,t)}^{\text{intravasating CTCs}} - \overbrace{r_{imm}\ CTC(t)\ \Gamma(\overline{C_{47}}-C_{47})}^{\text{CTCs do not escape the immune system}} +$$

$$- \overbrace{r_{ext}\ CTC(t)\ \Gamma(C_{44})\ \Gamma(C_{47})\ \Gamma(C_{MET})}^{\text{extravasating CTCs}} - \overbrace{r_{BP}\ BP(t)\ CTC(t)}^{\text{therapy effect}}$$

- CTC_e in the bone niche

$$\partial_t CTC_e(t) = \overbrace{r_{ext}\ CTC(t)\ \Gamma(C_{44})\ \Gamma(C_{47})\ \Gamma(C_{MET})}^{\text{extravasating CTCs}} - \overbrace{r_{seed}\ CTC_e(t)}^{\text{seeding CTCs}} - \overbrace{r_{BP}\ BP(t)\ CTC_e(t)}^{\text{therapy effect}}$$

- BP in the breast tissue, in the blood and in the bone

$$\partial_t BP(t) = \overbrace{H(t-t_1)\ H(t_2-t)\ \frac{\overline{BP}}{t_2-t_1}}^{\text{drug administration}} - \overbrace{d_{BP}\ BP(t)}^{\text{drug decay}} +$$

$$\overbrace{-\,r_{BP}\ BP(t)\ \left[\sigma_0 \sum_{\phi=1}^{\Phi}\rho(\phi,t) + CTC(t) + CTC_e(t)\right]}^{\text{drug absorption}}$$
