# Supplementary material for: Modelling Circulating Tumour Cells for Personalised Survival Prediction in Metastatic Breast Cancer
Source: PLoS Comput Biol. 2015 May 15;11(5):e1004199. doi: 10.1371/journal.pcbi.1004199 (PMC4433130; doi:10.1371/journal.pcbi.1004199)
Supplement: S3 Text — (PDF) [file pcbi.1004199.s003.pdf]

Modelling circulating tumour cells for personalised survival prediction in metastatic breast cancer

Gianluca Ascolani[1,◕], Annalisa Occhipinti[1,◕*], Pietro Liò[1,◕]

1 University of Cambridge, Computer Laboratory, Cambridge, UK

◕These authors contributed equally to this work. * ao356@cl.cam.ac.uk

S3 Text: Sensitivity Analysis

We performed a sensitivity analysis (SA) parameters checking to understand how parameter variations affects our model and how much these variations change the outputs. The SA allows us not only to identify those parameters whose perturbations have major effects on the model, but also to highlight the importance of parameters related to the treatment and to the branching process. The SA consists in computing the partial rank correlation coefficients (PRCC) between all the parameters and all the outputs. The PRCC is a measure concerned with the cases where there is a monotonic relationship between the single parameters and the outputs as in our model. The trajectories simulated were generated by sampling the parameters with the latin hypercube sampling methods (LHS). The multidimensional distribution used in the LHS was obtained by constraining each parameter to take values within a range of 10% variation from its nominal value with a uniform prior distribution, see the table with the list of parameters in the main text. We have computed the PRCC for different times before, during and after the administration of bisphosphonate, and we have selected those parameters whose p-values was smaller than 10^{-4}. In time, the importance and sensitivity of parameters changed; therefore, we have chosen a threshold $|PRCC| > 0.3$ as parameters significantly important to the dynamics. Source parameters for TGF-β in the breast compartment play a leading role in the evolution of the system showing that the different quantities of TGF-β produced by the mammary epithelial sub-population with different phenotypes is essential in the development of the disease. On the contrary, the exclusion of the TGF-β effects on the cellular population together with the cell capacity constraint would result in dynamics rapidly reaching a stationary state with quite no cancer cells. For this reason, we considered different datasets to estimate the values of $R^{\star}(\phi)$. In order to pinpoint the action of the treatment on the system, we reduced the range of variation for the parameters related to the TGF-β .S1-S6 Figures and S7-S12 Figures show the sensitivity analysis before and after the administration of the bisphosphonate.

CTCs and extravasating CTCs show the decay of the bisphosphonates d_{BP} which has a negative PRCC indicating that decreasing the time of permanence of the drug in the circulatory system favours the growing amount of the CTCs. On the contrary, the average quantity of drug injected $\overline{BP}$ is negatively correlated with cancer cells in the DLU and with both the CTCs and extravasating CTCs in the blood vessels. Extravasating CTCs are positively correlated to C_{44}, C_{47} and C_{MET}, while CTCs are negatively correlated to $\overline{C_{47}}$ showing how much CD47 is necessary for the CTCs to survive and to evade the immune system attacks. CD44, CD47 or MET alone are not sufficient to form CTCs, but the high expression of EPCAM is also required. The reason for this is that C_{EPC} helps cancer cells in the breast, which are free from strong cell-to-cell junctions, to intravasate the near blood vessels. The population of cells presenting overexpression of EPCAM, CD44, CD47 and MET are positively correlated with the probability u_0 of acquiring driver mutations. The action of bisphosphonate r_{BP} reduces the amount of cancer cells in the breast especially aggressive cancer cells. It also affects the number of CTCs.

As previously said the bisphosphonate directly act on the cancer cell reducing their proliferation and inducing apoptosis. The action of the bisphosphonates on the cancer cell populations in the three compartments

affects also the production of TGF-β in the respective environments and the action of the TGF-β on the neighbour cells as a cancer promoter. In our multi-compartmental model, we do not describe explicitly the TGF-β cycle (synthesis, activation, internalization), but the SA indicates that an increase in the parameters $R^{\star}(\phi)$ causes an increase of the cancer cell populations [1], and consequently, an increase of the TGF-β released.

References

1. Ascolani G, Liò P. Modeling TGF-β in Early Stages of Cancer Tissue Dynamics. PLoS ONE. 2014 02;9(2):e88533. Available from: http://dx.doi.org/10.1371%2Fjournal.pone.0088533.
