# Supplementary material for: Modelling Circulating Tumour Cells for Personalised Survival Prediction in Metastatic Breast Cancer
Source: PLoS Comput Biol. 2015 May 15;11(5):e1004199. doi: 10.1371/journal.pcbi.1004199 (PMC4433130; doi:10.1371/journal.pcbi.1004199)
Supplement: S4 Text — (PDF) [file pcbi.1004199.s004.pdf]

Modelling circulating tumour cells for personalised survival prediction in metastatic breast cancer

Gianluca Ascolani[1,◕], Annalisa Occhipinti[1,◕*], Pietro Liò[1,◕]

1 University of Cambridge, Computer Laboratory, Cambridge, UK

◕These authors contributed equally to this work. * ao356@cl.cam.ac.uk

S4 Text: Data Analysis

We have analysed a large number of breast cancer data sets (and other cancer types) available in public repositories with the aim to characterise the overexpression of CD47 and other markers (CD44, EPCAM) in ductal carcinoma in situ (DCIS) and invasive ductal carcinoma (IDC) versus control and in different experimental conditions. We did not focus explicitly on MET because its statistical behaviour is quite similar to the CD44 one.

We found that CD47 is not enriched in breast cancer with respect to other cancers (NCI cancer cell lines). In many datasets the p-value of CD47 is statistically non significant and often around 10^{-1}. The details about each dataset are shown in Table S1. CD47 does not turn statistically meaningful under tamoxifen (GSE28645); there is little or not increase of cancer stem cell population in highly metastatic variants of MDA-MB-231 breast cancer cell line (GSE25976 [66]). It is also not statistically significant in GDS3324 and in GSE6883 [67]. CD47 has not shown up in comparison cell lines from 9 different cancer tissue (NCI-60) (U95 platform)(GSE5949, GSE6691 [68])

Despite in all the sample the (non adjusted) p-value is not statistically meaningful, the relatively constant value around 10^{-1} is consistent with a high heterogeneity of CD47 expression: most cancer cells have CD47 expression similar to non cancer cells; only few have higher values of CD47 gene expression. Perhaps these cells with high CD47 expression are under strong selection because they are at the boundaries of the cancer and therefore being more challenged by the immune response than the other cancer cells. The fact that EPCAM has usually more statistically significant values than CD47 is in agreement with this hypothesis.

References

1. Okuda H, Kobayashi A, Xia B, Watabe M, Pai SK, Hirota S, et al. Hyaluronan Synthase HAS2 Promotes Tumor Progression in Bone by Stimulating the Interaction of Breast Cancer Stem–Like Cells with Macrophages and Stromal Cells. Cancer research. 2012;72(2):537–547.

2. Planche A, Bacac M, Provero P, Fusco C, Delorenzi M, Stehle JC, et al. Identification of prognostic molecular features in the reactive stroma of human breast and prostate cancer. PloS one. 2011;6(5):e18640.

3. Pfister TD, Reinhold WC, Agama K, Gupta S, Khin SA, Kinders RJ, et al. Topoisomerase I levels in the NCI-60 cancer cell line panel determined by validated ELISA and microarray analysis and correlation with indenoisoquinoline sensitivity. Molecular cancer therapeutics. 2009;8(7):1878–1884.
