# Supplementary material for: Modelling Circulating Tumour Cells for Personalised Survival Prediction in Metastatic Breast Cancer
Source: PLoS Comput Biol. 2015 May 15;11(5):e1004199. doi: 10.1371/journal.pcbi.1004199 (PMC4433130; doi:10.1371/journal.pcbi.1004199)
Supplement: S1 Table — Details of the datasets analysed to estimate the p-value of overexpression of CD47, CD44 and EPCAM in ductal carcinoma in situ (DCIS) and invasive ductal carcinoma (IDC) versus control under different experimental conditions. (PDF) [file pcbi.1004199.s005.pdf]

Modelling circulating tumour cells for personalised survival prediction in metastatic breast cancer

Gianluca Ascolani[1,◕], Annalisa Occhipinti[1,◕*], Pietro Liò[1,◕]

1 University of Cambridge, Computer Laboratory, Cambridge, UK

◕These authors contributed equally to this work. * ao356@cl.cam.ac.uk

S1 Table. List of datasets

Accession Number	Reference	p-values CD47	EPCAM	CD44
GSE29044	Colak et al. [69]	4.39^{-02}	1.17^{-03}	6.70^{-02}
GSE15852	Pau et al. [70]	1.05^{-01}	$4.88\ 10^{-17}$	4.49^{-02}
GSE55070	Drews et al. [71]	1.18^{-01}	N/A	N/A
GSE46563	Jonsdottir et al. [72]	0.11326064	0.41234569	0.00432464
GSE54465	Limame et al. [73]	1.01^{-02}	5.66^{-01}	1.1390259
GSE46141	Kimbung et al. [74]	0.489669	0.125039	0.89891
GSE48371	Pagliuca et al. [75]	0.1558076	0.323313	0.0980182

Details of the datasets analysed to estimate the p-value of overexpression of CD47, CD44 and EPCAM in ductal carcinoma in situ (DCIS) and invasive ductal carcinoma (IDC) versus control under different experimental conditions.
